# Improved GPS tropospheric path delay estimation using variable random walk process noise

**DOI:** 10.1007/s00190-024-01898-3

**Published:** 2024-10-07

**Authors:** Zachary M. Young, Geoffrey Blewitt, Corné Kreemer

**Affiliations:** 1 Nevada Bureau of Mines and Geology, University of Nevada, 1664 N Virginia St. MS 178, Reno, NV 89557 USA; 2 Department of Geosciences, University of Montana, 32 Campus Dr., Missoula, MT 59812 USA

**Keywords:** GPS, Tropospheric delay, Estimation strategy, Random walk, Optimization, GipsyX

## Abstract

Accurate positioning using the Global Positioning System relies on accurate modeling of tropospheric delay. Estimated tropospheric delay must vary sufficiently to capture true variations; otherwise, systematic errors propagate into estimated positions, particularly the vertical. However, if the allowed delay variation is too large, the propagation of data noise into all parameters is amplified, reducing precision. Here we investigate the optimal choice of tropospheric constraints applied in the GipsyX software, which are specified by values of random walk process noise. We use the variability of 5-min estimated positions as a proxy for tropospheric error. Given that weighted mean 5-min positions closely replicate 24-h solutions, our ultimate goal is to improve 24-h positions and other daily products, such as precise orbit parameters. The commonly adopted default constraint for the zenith wet delay (ZWD) is 3 mm/√(hr) for 5-min data intervals. Using this constraint, we observe spurious wave-like patterns of 5-min vertical displacement estimates with amplitudes ~ 100 mm coincident with Winter Storm Ezekiel of November 27, 2019, across the central/eastern USA. Loosening the constraint suppresses the spurious waves and reduces 5-min vertical displacement variability while improving water vapor estimates. Further improvement can be achieved when optimizing constraints regionally, or for each station. Globally, results are typically optimized in the range of 6–12 mm/√(hr). Generally, we at least recommend loosening the constraint from the current default of 3 mm/√(hr) to 6 mm/√(hr) for ZWD every 300 s. Constraint values must be scaled by √(*x*/300) for alternative data intervals of *x* seconds.

**Supplementary Information:**

The online version contains supplementary material available at 10.1007/s00190-024-01898-3.

## Introduction

Estimates of Global Positioning System (GPS) station positions, at 24-h static and sub-daily kinematic data rates, have a broad variety of scientific applications, including analyzing early co-/post-seismic mechanisms and earthquake early warning systems (Colombelli et al. 2013; Larson 2009; Melgar et al. 2012; Twardzik et al. 2019), atmospheric applications such as integrated water vapor (IWV) and total electron content (TEC) (Fu et al. 2021; Leontiev and Reuveni 2017; Moore et al. 2015; Rocken et al. 1997; Sun et al. 2021), and real-time volcano monitoring (Escayo et al. 2020; Larson et al. 2010). For well over a decade, the Nevada Geodetic Laboratory (NGL) has routinely produced both 24-h and 5-min station position estimates for all publicly available geodetic GPS data (Blewitt et al. 2018). The 5-min solutions are produced by NGL mainly to enable determination of earthquake mechanisms from the permanent displacement of GPS stations that occurs within a daily data set (e.g., Blewitt et al. 2006, 2009), but also to provide additional quality statistics for each day.

Such applications require quality positions and consistent repeatability of the 5-min position estimates to distinguish true station motion. While useful in themselves, 5-min position solutions can also be used as a tool to probe and reduce systematic errors in the GPS observable model, particularly with regard to tropospheric delay and related parameter estimation strategy. As this paper demonstrates, when the scatter of 5-min position estimates is minimized, the more widely applied 24-h static positions exhibit improved stability, thus improving the analysis of longer-term geophysical signals.

The topic of this paper is on improving station coordinate estimation, particularly in the vertical, through improved estimation of time-variable delay in the troposphere. For example, of concern is the ability of the troposphere estimation strategy to capture variations in delay arising from rapidly evolving weather fronts (Gregorius and Blewitt 1999; Luddington et al. 2010). Tropospheric delay models are conventionally separated into a so-called dry delay (~ 2 m) and a wet delay (~ 0.1 m). The wet delay is caused by the interaction of the electromagnetic (EM) waves with the permanent dipole moment of molecules of water vapor. The dry delay is caused by the interaction of EM waves with the induced dipole moments of all gasses in the atmosphere (including a “dry” component from water vapor). In hydrostatic equilibrium, the dry delay relates to air pressure which is typically slowly varying and well modeled (Saastamoinen 1973). However, the wet component relates to water vapor, which can be highly variable and much more difficult to model (Bar‐Sever et al. 1998).

To model tropospheric dry and wet delay, tropospheric mapping functions are used to map zenith dry delay (ZDD) and zenith wet tropospheric delay (ZWD) to the elevation angle of the satellite being observed. Thus, all wet delays can be modeled by estimating a bias in the ZWD. Moreover, the bias in ZWD can be estimated stochastically to allow for time variation (Tralli and Lichten 1990). More accurate models introduce two additional parameters to account for a 2D gradient in tropospheric delay (Bar-Sever et al. 1998). This approach is implemented in the GPS data analysis software “GipsyX” from the Jet Propulsion Laboratory (JPL), which is used for this study (Bertiger et al. 2020).

Note that residual errors in ZDD will be almost entirely absorbed by the estimate of ZWD; therefore, GPS is mostly sensitive to zenith total delay (ZTD), where ZTD = ZDD + ZWD. For the purpose of accurate positioning (the objective of this paper), accurate estimation of ZTD is relevant. For the purpose of accurate estimation of IWV, accurate estimation of ZWD is relevant.

Current models of the zenith dry and wet mapping functions are based on numerical weather models (NWM). For example, the Vienna Mapping Function Model 1 (VMF1) (Boehm et al. 2009), which is implemented in GipsyX, is based on the NWM of the European Center for Medium Range Weather Forecasting (ECMWF). The VMF1 mapping functions are produced on a spatial grid in 6-h intervals, which are then interpolated to the required locations and epochs. Using GipsyX, the ZWD estimate is estimated stochastically (allowing ZWD to vary at each 5-min epoch), while being constrained by random walk process noise. Following Bar‐Sever et al. (1998), the default 1-sigma value in GipsyX for this process noise is 3 mm/√(hr) for the zenith delay and 0.3 mm/√(hr) for the two (east and north) horizontal gradients. Values approximately equivalent to these have often been used by studies (e.g., Geng et al. 2012; Sun et al. 2021; Xu et al. 2013) and GPS processing centers (e.g., Blewitt et al. 2018; Herring et al. 2016). If the real ZTD experiences a significant change with time, however, a strict random walk constraint would limit variability of the ZTD estimate, and the additional displacement will propagate into the position of the GPS station. As weather fronts pass through a region, atmospheric moisture (and to some extent, atmospheric pressure) can vary rapidly, resulting in large, rapid variations in the real ZTD (Gregorius and Blewitt 1999; Luddington et al. 2010).

**Fig. 1:**
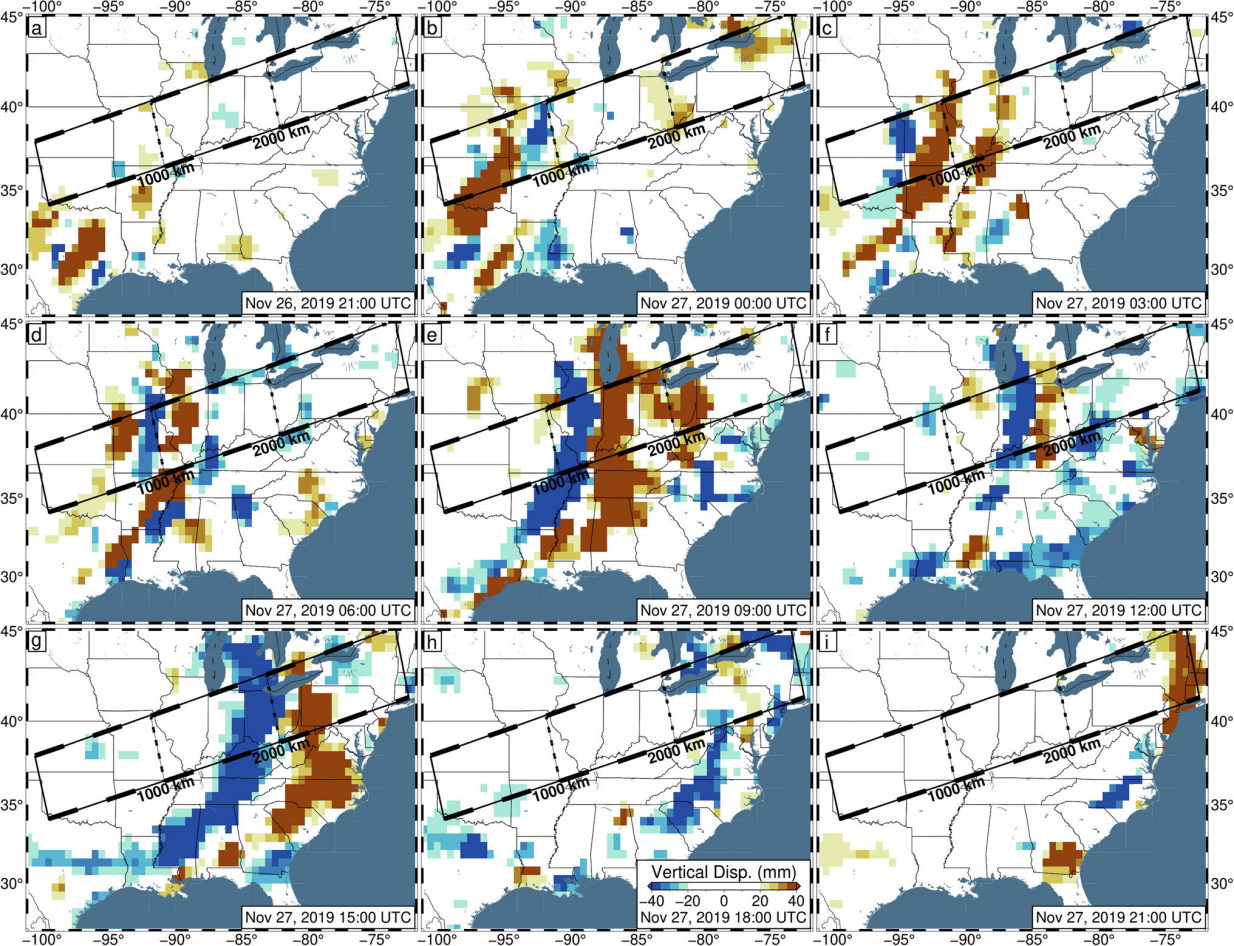
Observed 5-min GPS vertical displacements in 3-h intervals between 21:00 UTC on November 26, 2019, and 21:00 UTC on November 27, 2019. Data have been filtered with Robust Network Imaging (Kreemer et al. 2020) and are produced using the current NGL data analysis strategy, with the default ZWD random walk constraint of 3 mm/√(hr). The color bar is set such that colors begin near the average global vertical position uncertainty and saturate at two sigma. Reds indicate observed uplift and blues reflect observed subsidence. The black rectangle represents the bounds of the transect shown in Figs. 2, 8, and S3. Line segments along the length of the transect each represent 250 km of distance

**Fig. 2:**
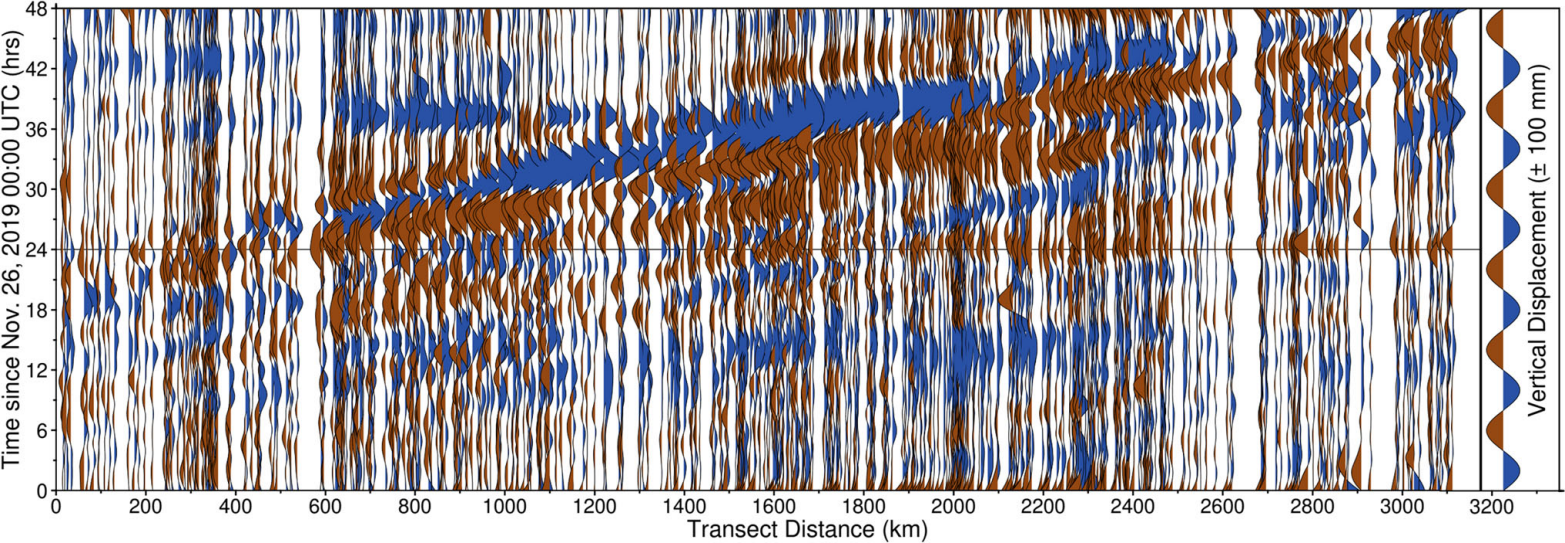
Wiggle plot of observed GPS vertical displacements along the transect shown in Fig. 1 for November 26–27, 2019. The transect begins in western Oklahoma and culminates in Vermont. Data have been smoothed with robust weighted local regression (RLOESS), with a smoothing factor of 0.10. Black horizontal bar represents 00:00 UTC on November 27. Note the systematic wave-like displacements which propagate across the entirety of the transect on November 27

For example, preliminary inspection of 5-min vertical GPS positions across the central/eastern USA on November 26–27, 2019 UTC, reveals significant apparent displacements. These dates mark the regional onset of Winter Storm Ezekiel, which produced significant blizzard conditions across the entirety of the USA (LeComte 2020). Not only are the displacements large (~ 100 mm), but they also form coherent bands of uplift/subsidence which travel ~ 3000 km from Texas to Maine throughout the day (Fig. 1, Video S1). When observed in transect (Fig. 2), these displacements appear wavelike, with a consistent propagation speed. While studies have linked atmospheric pressure loading to GPS displacements (Martens et al. 2020; Tregoning and van Dam 2005), the expected atmospheric loading displacements caused by the storm are too broad, and of low magnitude (less than 10 mm spread over the day) (GFZ 2022), to explain the regionally variable displacements. We hypothesize that the apparent displacements are artifacts of tropospheric mismodeling caused by the (default) random walk constraints being overly strict.

**Fig. 3:**
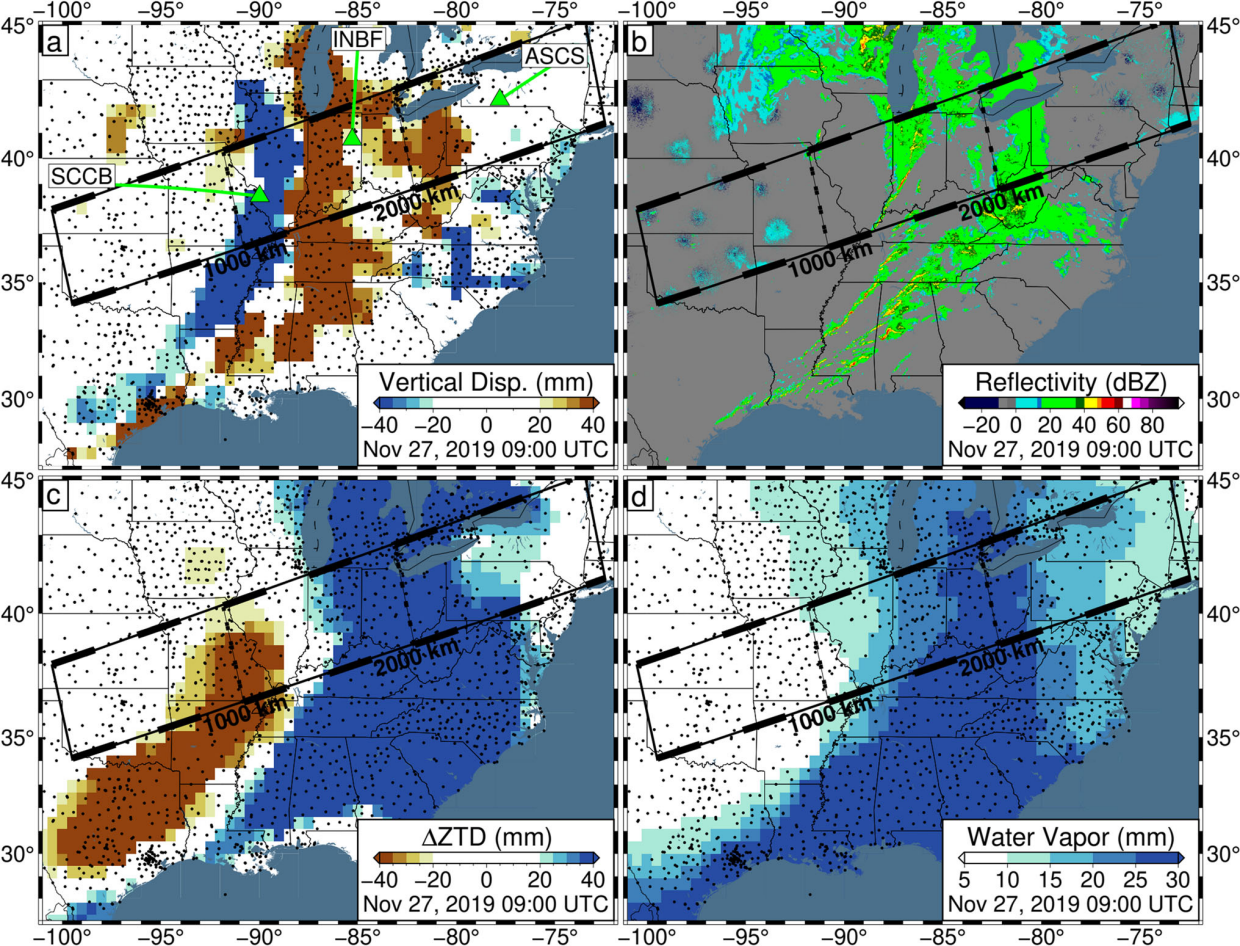
Data snapshots on November 27, 2019, at 09:00 UTC for the central/eastern USA. **a** Observed GPS vertical displacements. Green triangles represent the locations of GPS stations SCCB, INBF, and ASCS whose time series are presented in Fig. 4. **b** Radar reflectivity (green values indicate precipitation; Herzmann 2022). **c** Zenith total delay (ZTD) deviation. For each station, the median ZTD across November 26–27 is removed. **d** Integrated water vapor inferred by GPS. Note the spatial correlation between vertical uplift and observed radar reflectivity. Data presented in panels a, c, and d are produced using the current NGL data analysis strategy and have been filtered with Robust Network Imaging. Small black dots represent GPS station locations

The observed vertical displacements, and their progression, are spatially correlated with radar reflectivity maps throughout the day (Herzmann 2022). This is highlighted in Fig. 3 which provides a comparison of these observations at 09:00 UTC on November 27, 2019. The radar reflectivity map reveals the presence of a wide band of precipitation across the study area, which strongly matches the distribution of vertical uplift observed by GPS. Additionally, the western edge of the precipitation matches the boundary between uplift and subsidence and steep gradients in both the ZTD and integrated water vapor. A primary driver of precipitation is the interaction of warm and cold air masses. As a cold air mass moves under the warm air mass, it pushes moisture higher into the atmosphere allowing it to condense as it cools, forming precipitation. The spatial correlation between the observations suggests that the vertical GPS displacements are dominated by errors related to atmospheric variability associated with the weather fronts of the storm.

Gregorius and Blewitt (1999) investigated the impact of weather fronts on 24-h estimates of vertical GPS positions. Their results revealed that vertical repeatability was significantly improved when considering a ZWD random walk constraint of 8 mm/√(hr) on days when fronts passed over the stations. That study, however, was limited to 21 globally distributed stations, preventing substantial investigation into regional and climatic aspects of varying the constraint. Additionally, Penna et al. (2015) found an optimal ZWD constraint of 6 mm/√(hr) when investigating tidal displacements in kinematic GPS time series.

In the last 10-20 years, GPS data processing methods have greatly improved and the global GPS network has experienced significant expansion (Blewitt et al. 2018), improving the ability to distinguish the sensitivity of 5-min GPS positions to the chosen random walk constraint, at both local and global scales. In this study, several suites of 5-min GPS time series are produced, featuring different levels of ZTD random walk constraints, and we investigate both its impact on vertical positions during Winter Storm Ezekiel, as well as its effect on global data quality. We then provide recommendations for future processing strategies to improve both 5-min kinematic and 24-h static GPS positioning quality.

## Methods

### Data analysis strategy using GipsyX

Except where noted, this investigation uses JPL’s GipsyX software (Bertiger et al. 2020) with NGL’s standard production data analysis strategy for kinematic precise point positioning in the IGS14 reference frame. The strategy is based on the precise point positioning concept by Zumberge et al. (1997) with fixed satellite orbits and clock biases, but with station coordinates estimated freely at every 5-min epoch. GPS satellite positions and clocks are from JPL’s “final” product line. Batch solutions are generated for 24-h GPS days for each station individually using undifferenced carrier phase and pseudorange data for every 5-min epoch from all satellites in view. Elevation angles are limited to *e* >  = 7°, with observation weights that scale with sin(*e*). Station clock biases and station coordinates are estimated with loose constraints so that they are essentially data driven.

Positions which exhibit a 3-dimensional formal error greater than 0.1 m are rejected. This guards against poor data during periods of time where there may be poor observability and geometry of the GPS satellite constellation. But otherwise, outliers are not rejected based on their estimated values, because they may faithfully represent actual geophysical motion, such as co-seismic displacement.

The formal errors for 5-min station positions are given by GipsyX in the global Cartesian coordinate system. These are transformed into the topocentric coordinate system (east, north up) assuming the approximation of zero correlation between east, north, and up, and assuming the ratio of variances between east:north:up is 0.55:0.80:8.00, a generally good approximation that was inferred by full covariance analysis. Then, the formal errors are scaled by a factor of 2, resulting in formal errors of 24-h weighted average 5-min coordinates that agree closely with the level of formal errors and full covariances of coordinates estimated directly (as constants) every 24 h. (Effectively, the scaling factor of 2 accounts for formal temporal correlations in the 5-min coordinates that are ignored when taking the weighted average).

Ionospheric delay is calibrated using a linear combination of dual-frequency GPS data and is modeled to higher order using a conventional model of the Earth's magnetic field combined with JPL data products on ionospheric total electron content. As outlined in the introduction, tropospheric modeling uses VMF1 data products (Boehm et al. 2009) for ZDD and ZWD, and for dry and wet mapping function parameters. Residual ZTD variation is estimated as a random walk in ZWD with a default constraint of 3 mm/√(hr) for the zenith delay and 0.3 mm/√(hr) for the two horizontal gradients (Bar-Sever et al. 1998). The overall level (starting value) of residual ZTD is estimated very loosely with a 0.5 m constraint. Carrier phase bias parameters are introduced for each station-satellite arc and for detected integer discontinuities, some of which may be automatically resolved prior to least-squares estimation (Blewitt 1990). All parameters (typically about 70 per station day) are estimated simultaneously using a square root information filter. GipsyX then constrains real-valued estimates of carrier phase biases with the a priori knowledge that they are linear combinations of integer wavelengths (Blewitt 1989; Bertiger et al. 2010). This improvement is propagated through to all parameters, including the 5-min tropospheric parameters and 5-min positions.

### Rationale to improve solutions by reducing scatter of positions

Reducing the repeatability of 24-h solutions has long been used as an indicator of improvement in modeling and estimation strategy since the early days of GPS, e.g., for improved orbit determination (Lichten and Border 1987), improved troposphere estimation (Tralli and Lichten 1990), and the efficacy of ambiguity resolution (Dong and Bock 1989; Blewitt 1989; Bertiger et al. 2010). One might therefore reasonably hypothesize that reducing the root-mean-square (RMS) scatter of 5-min positions may also lead to improved solutions.

Indeed, it has been shown by Blewitt et al. (2015) that the weighted mean of 24-h of 5-min solutions exhibits negligible differences ~ 0.1 mm in daily RMS scatter when compared to the RMS of standard 24-h static positions. In accordance with least-squares theory, the rigorous combination of blocks of estimated parameters into a reduced set of parameters (in this case, from multiple 5-min positions into one 24-h position) leads to identical solutions as estimating the reduced set of parameters directly. This is the basis of many efficient block partitioning schemes in least squares (Blewitt 1998). In fact, this is how NGL routinely produces rapid 24-h solutions (Blewitt et al. 2018), rather than wastefully reprocessing the data a second time to estimate a single 24-h position. As such, the 5-min positions presented in this study provide direct insight into the quality of the static 24-h solutions, as reducing the scatter of 5-min solutions improves the repeatability of daily solutions. Moreover, we could directly use the weighted mean 5-min solutions in the place of traditional 24-h solutions without loss of precision to assess quality. Nevertheless, this paper will use traditional 24-h solutions to assess quality as evidence to support our rationale.

Analysis of the 5-min kinematic position estimates used in this study provides key benefits over the more traditional 24-h static solutions, such those used by Gregorius and Blewitt (1999). The first is that by investigating the 5-min solutions, we open the analysis for a wider range of statistical methods and improve their robustness. Another benefit is that we are able to observe the evolution of the impact of the weather systems on the positions before, during, and after, which typically occurs over the time frame of a few hours and is difficult to distinguish when viewing the more traditional 24-h static solutions.

Ultimately, our goal is to find an optimal estimation strategy to improve the more widely used static 24-h positions. Moreover, since precise orbit determination (Lichten and Border 1987) also involves estimation of all 24-h station positions in the global GPS network (Heflin et al. 1992), it therefore follows that precise orbit parameters should also improve, thus leading to improved general GPS positioning when using such precisely estimated orbits, such as using precise point positioning (Zumberge et al. 1997; Bertiger et al. 2010). Although beyond the scope of this paper, if such improvements were implemented in routine orbit determination, this would then have a truly broad impact on the precise GPS positioning community.

### Proxy for tropospheric errors

Here we use the 5-min vertical GPS position variations as a proxy for errors in ZTD estimation.

This proxy method assumes that errors in ZTD caused by rapid variation (that exceed the random walk constraints) will generate much larger vertical position errors (e.g., 10–100 mm or more over a few hours) than real vertical displacement from atmospheric pressure and surface water loading (e.g., under 10 mm over several hours/days). More generally, we consider this also a proxy for the more traditionally estimated station positions as constant over each 24-h GPS day, for which our assumption is that 24-h static position estimates are optimized (have the least error) when 5-min vertical position estimates have the smallest variation.

### Rationale for loosening random walk constraints

As a preliminary study, we investigated vertical GPS positions across the central/eastern USA on November 26–27, 2019 UTC. These days are chosen to investigate the regional impact of the significant atmospheric turbulence produced by Winter Storm Ezekiel which initiated toward the end of the day on November 26 and traversed the entire study area on November 27.

If the default random walk constraint of 3 mm/√(hr) is appropriate, and the observed displacements are associated with an un-modeled geophysical signal, then loosening the random walk will amplify the mapping of measurement noise into the 5-min vertical positions (due to correlations between vertical and ZTD parameters). This would increase station 5-min vertical coordinate RMS scatter while preserving the original signal. However, if the random walk constraint is too tight, loosening its value will allow the ZTD estimate to better account for atmospheric variability, and errors in 5-min vertical positions should decrease as systematic error is reduced. This would reduce station RMS and suppress the apparent displacements.

To further investigate the random walk constraints under more typical conditions, we then expand our study to the global network and explore the regional and temporal aspects of loosening the constraints. We also look at the effect of loosening constraints on 24-h static estimates of vertical coordinates, which is of interest for investigations of slow deformations of the Earth.

### Test tropospheric estimation strategies

To address the above goals, daily batches of 5-min kinematic and 24-h static GPS vertical data are produced for November 26–27, 2019 UTC, as well as for the first two days of each month of 2021. We adhere to the NGL standard data analysis strategy (Sect. 2.1) but with a suite of test estimation strategies for which we increase the random walk constraint for ZWD incrementally between the default value of 3 mm/√(hr) and 48 mm/√(hr). Table 1 shows the constraints used for the specified test estimation strategies. Note that the horizontal gradient constraints are equivalently increased, at a value that is 10% of the zenith constraint. The gradient process noise is a second-order effect compared to the zenith process noise; thus, we retain the ratio presented by Bar-Sever et al. (1998) and do not test this ratio in this study. We emphasize that for our findings to be more generally useful, these process noise values may be scaled for alternate data rates by a factor of √(*x*/300), where *x* is the chosen data interval in seconds.

**Table 1:** Specified 5-min kinematic and 24-h static data rate test estimation strategies

		Random walk constraint [mm/√(hr)]		
Strategy name	Strategy ID	Zenith wet delay	Gradient east	Gradient north
TROPx01*	01	3	0.3	0.3
TROPx02	02	6	0.6	0.6
TROPx04	04	12	1.2	1.2
TROPx08	08	24	2.4	2.4
TROPx12	12	36	3.6	3.6
TROPx16	16	48	4.8	4.8
TROPxCS	CS	**	**	**
TROPxOP	OP	***	***	***

^*^Default strategy in GipsyX (Bar-Sever et al. 1998)

^**^Characteristic station specific value

^***^Daily optimal station specific value

Henceforth, we will refer to specified strategies either by its full name or identification (ID) number, where ID is the scaling factor of the default strategy. These test strategies generate our “uniform” random walk solutions, in the sense that each strategy is applied to all stations for all days tested without attempt to optimize based on location or time. For the purpose of this study, we focus on the results of the vertical component as our proxy for tropospheric error. Estimation of all other parameters is kept at the default settings (Sect. 2.1), thus isolating the impact of loosening the random walk constraints of the tropospheric parameters.

In addition to the uniform random walk data sets, two data sets are produced in which the random walk is allowed to vary per station. The first, our “characteristic station specific” (CS) data set, identifies the strategy which most frequently minimizes the RMS of 5-min vertical estimates for each station during 2021. This method allows for spatial variability in the distribution of station random walk constraints. The second, our “daily optimal” (OP) data set, identifies the solution which minimizes the RMS 5-min vertical for each station, on each day, producing a spatio-temporally variable random walk data set. For consistency within the global analysis, we limit stations to those who produced data on all 24 days of our study, resulting in 5819 stations and 139,656 station days.

## Results

### Loosening constraints for Winter Storm Ezekiel

**Fig. 4:**
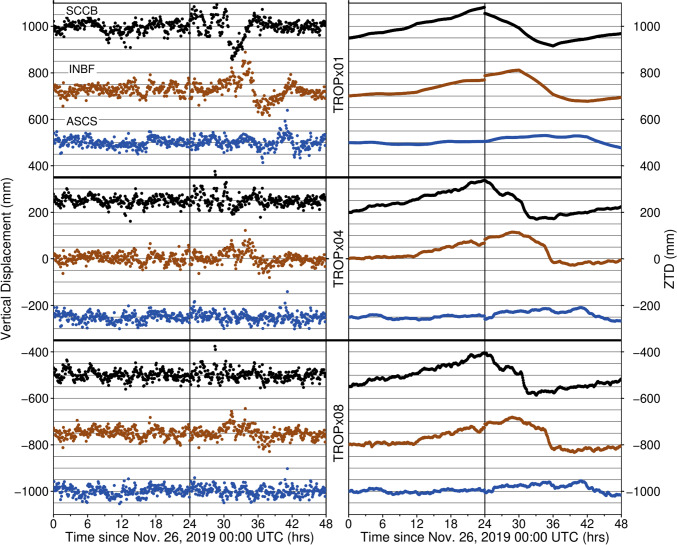
Comparison of vertical displacement and zenith total delay (ZTD) for GPS stations SCCB (black), INBF (red), and ASCS (blue) for three processing strategies. Station locations are shown in Fig. 3 and are distributed along the transect to highlight the progression of the storm. Vertical black line identifies the change of day

Loosening the random walk constraints clearly improves vertical data quality at stations across the central/eastern USA on November 26–27, 2019 UTC. Figure 4 shows a comparison of vertical displacements and ZTD estimates for GPS stations SCCB, INBF, and ASCS. The locations of these stations are shown in Fig. 3. On November 26, station positions are consistent, exhibiting only minor variation associated with the initiation of the storm. However, on November 27, displacements of up to 140 mm are observed as the storm intensifies and progresses eastward. Across both days, the TROPx01 ZTD estimates are smooth. Upon loosening the random walk constraint to 12 mm/√(hr) (four times looser, strategy TROPx04), the observed vertical deviations associated with the storm are reduced and the station ZTD estimate becomes more variable. The displacements associated with the storm are further suppressed when applying the TROPx08 strategy, with SCCB and ASCS showing no clear variation above the data noise and INBF being mostly corrected.

While the signal from the storm shows more improvement with TROPx08, station scatter begins to increase. This is shown through the 5-min vertical repeatability values presented in Table 2. Here, we define repeatability as 1.4826*MAD (Huber 1981), where MAD is the median absolute deviation of the time series (Hampel 1974). We see that station SCCB shows the best repeatability on November 27, with the TROPx08 solution, INBF is most improved with the TROPx04 solution, and ASCS is optimized when using the TROPx02 solution. While each station exhibits a different optimal strategy, repeatability values for all strategies are substantially lower than obtained with the TROPx01 solution. Although atmospheric conditions were calmer for most of the day on November 26, these stations show improvements with the TROPx02 and TROPx04 strategies. Notably, comparison between the optimal solutions for each day per station reveals similar levels of RMS values on both days when the constraint is allowed to vary.

**Table 2:** Five-min vertical repeatability by strategy for the stations presented in Fig. 4

	Strategy ID					
	01	02	04	08	12	16
Station	Station 5-minute vertical repeatability (mm)					
*November 26, 2019*						
SCCB	21.6	18.5	*17.4	20.7	22.3	23.7
INBF	20.3	20.3	*19.8	20.5	20.4	19.2
ASCS	17.4	*16.6	17.6	19.4	21.7	24.5
*November 27, 2019*						
SCCB	30.3	25.9	23.9	*19.5	20.7	23.3
INBF	37.3	28.6	*20.1	21.3	21.6	22.2
ASCS	21.7	*17.4	17.8	20.5	21.5	22.7

^*^Optimal solutions per station and day

**Fig. 5:**
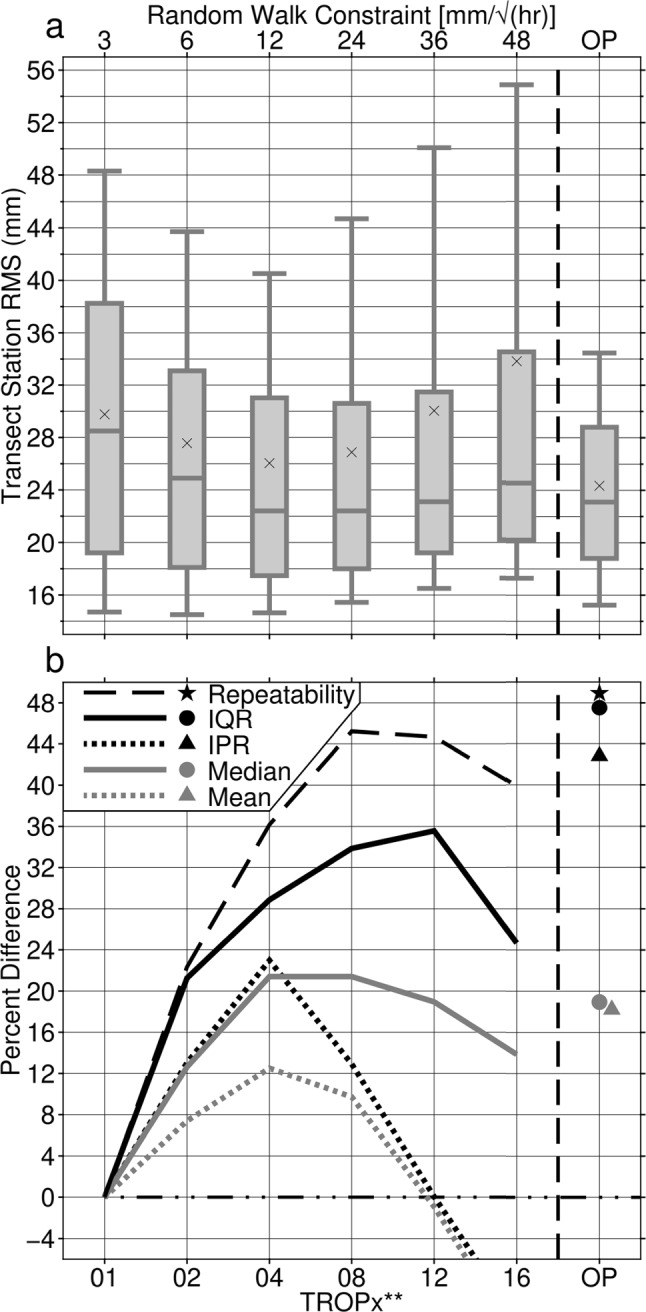
Data quality comparison by processing strategy for GPS stations along the transect presented in Figs. 1 and 2, on November 27, 2019. **a** Box and whisker plot of station RMS. The boxes represent the second and third quartiles with their width representing the inter-quartile range (IQR). The median is represented by the central line and the mean is denoted by an x. The whiskers represent the inter-percentile range (IPR) between the 10th and 90th percentiles. **b** Percent difference relative to the TROPx01 solution for station repeatability (black dashed line, black star), IQR (solid black line, black circle), IPR (dotted black line, black triangle), median (solid grey line, grey circle), and mean (dotted grey line, grey triangle). Vertical dashed line separates uniform strategies on the left from the variable daily optimal (OP) strategy on the right. Horizontal dash-dotted black line represents equivalence to the TROPx01 solution. Positive percent difference values indicate improvement

The passage of Winter Storm Ezekiel greatly affected regional vertical data quality. Figure 5 shows a comparison of the distribution of station 5-min vertical RMS along transect for different processing strategies and the percent difference of several representative statistics relative to the TROPx01 solution. Here the boxes denote the second and third quartiles [i.e., the inter-quartile range (IQR)], while the whiskers indicate the 10th and 90th percentiles of the distributions [i.e., the inter-percentile range (IPR)]. Large improvements to the distribution of station RMS values are observed with the TROPx02, 04, and 08 strategies relative to the TROPx01 solution; however, beginning with the TROPx08 solution, scatter due to increased measurement noise begins to increase rapidly as the constraint is loosened further. The median, mean, and IPR of the distributions are most improved with the TROPx04 solution at 21%, 13%, and 23%, respectively (Table 3). Station repeatability is most improved with the TROPx08 and 12 solutions at 45%, and the IQR sees 36% improvement with the TROPx12 strategy. Larger improvements are observed when the constraint is allowed to be optimized per station and the distribution of station RMS becomes much more normally distributed. The OP strategy obtains 49% improvement in station repeatability, and the IQR and IPR improve by 48% and 43%, respectively. By allowing variability in the constraint, stations which already exhibited low RMS values and were not significantly affected by the storm are not influenced by additional measurement noise introduced by loosening the constraint. On the other side of the distribution, where stations are affected by the storm to varying degrees, variability in the constraint allows for an improved balance between correcting errors associated with the storm and reducing the influence of measurement noise.

**Table 3:** Station RMS statistics by strategy along transect on November 27, 2019, and their percent difference relative to the TROPx01 solution

	IQR (mm)	IPR (mm)	Median (mm)	Mean (mm)	Repeatability (mm)
TROPx01	19.1	33.6	28.5	29.8	13.9
TROPx02	15.0	29.2	24.9	27.6	10.8
TROPx04	13.6	25.9	22.4	26.0	8.9
TROPx08	12.6	29.3	22.4	26.9	7.6
TROPx12	12.3	33.6	23.1	30.0	7.7
TROPx16	14.4	37.6	24.6	33.8	8.4
TROPxOP	10.0	19.2	23.1	24.3	7.1
*Percent difference relative to the TROPx01 solution*					
TROPx02	21	13	13	7	22
TROPx04	29	23	21	13	36
TROPx08	34	13	21	10	45
TROPx12	36	0	19	− 1	45
TROPx16	25	− 12	14	− 14	40
TROPxOP	48	43	19	18	49

**Fig. 6:**
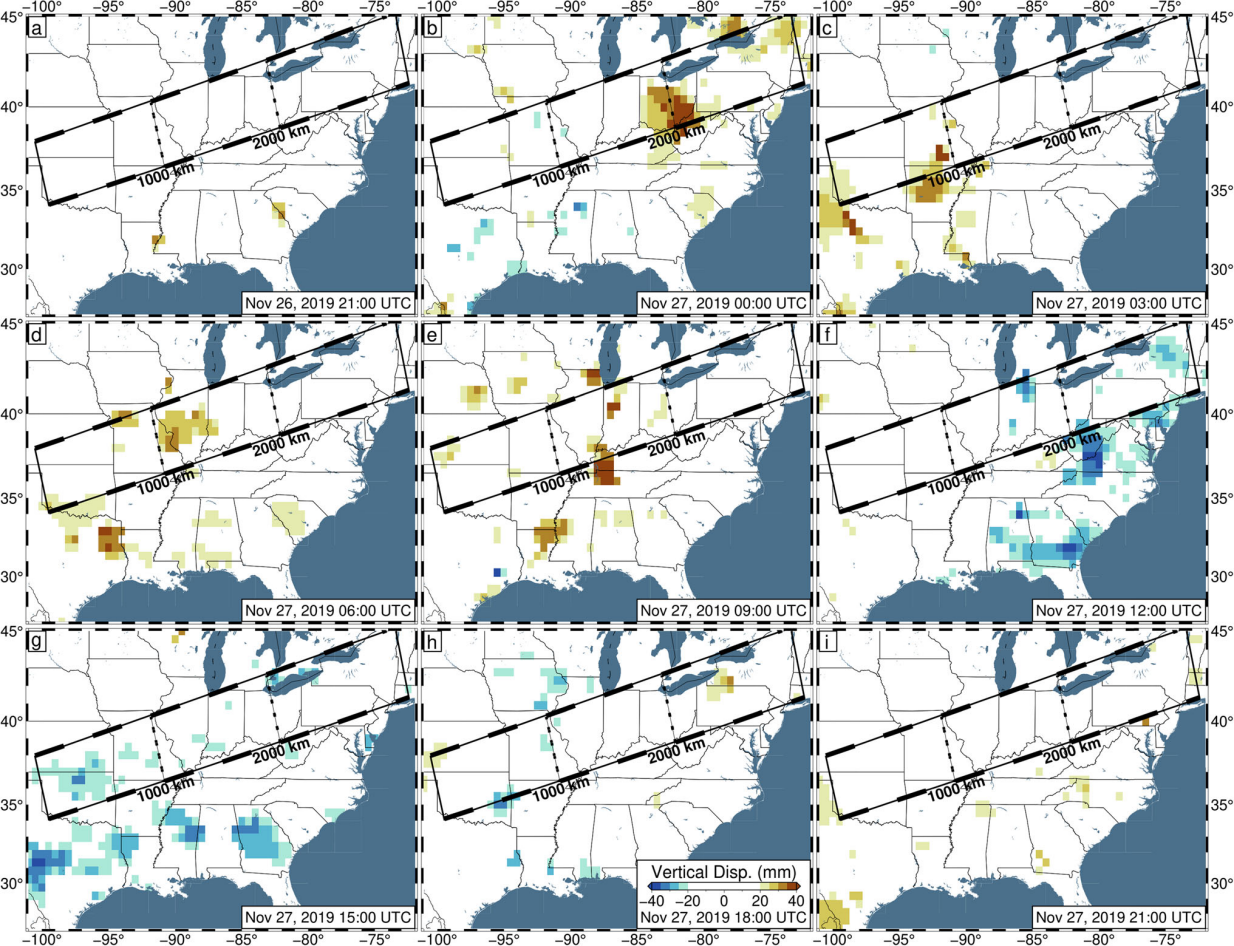
TROPx08 solution 5-min GPS vertical displacements in 3-h intervals between 21:00 UTC on November 26, 2019, and 21:00 UTC on November 27, 2019. Key as described in Fig. 1. Note that regionally systematic displacements have been suppressed

**Fig. 7:**
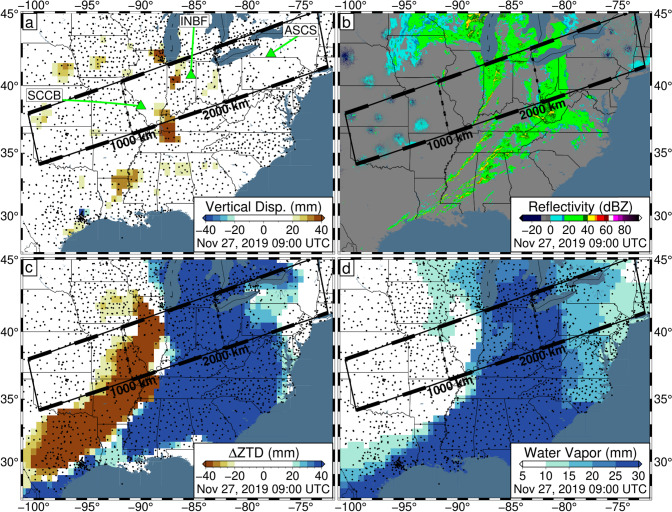
Same as Fig. 3 except for the TROPx08 solution [24 mm/√(hr)]. Note that vertical displacements are now suppressed and the spatial correlation of the ZTD and IWV estimates, to the radar reflectivity, is increased

**Fig. 8:**
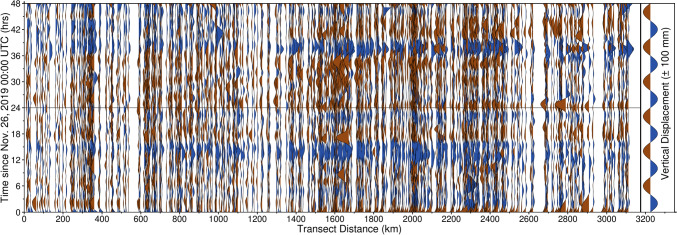
Wiggle plot of GPS vertical displacements along the transect identified in Fig. 1 for November 26 – 27, 2019, for the TROPx08 solution [24 mm/√(hr)]. Data have been smoothed with RLOESS regression, with a smoothing factor of 0.10. Black horizontal bar represents November 27 00:00 UTC. Note that the wave-like systematic displacements shown in Fig. 2 are suppressed. The subsidence signals observed at 15 h and 39 h are due to an increase in the regional common mode and are present across all solutions for most stations within North America

**Fig. 9:**
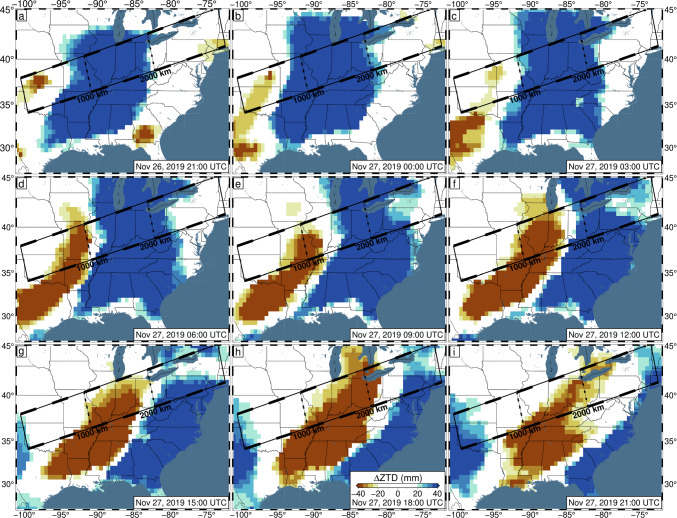
Observed zenith total delay deviation, in 3-h intervals between 21:00 UTC on November 26, 2019, and 21:00 UTC on November 27, 2019. For each station, the median delay across November 26–27 is removed. Data have been filtered with Robust Network Imaging and are produced using a random walk constraint of 3 mm/√(hr)

**Fig. 10:**
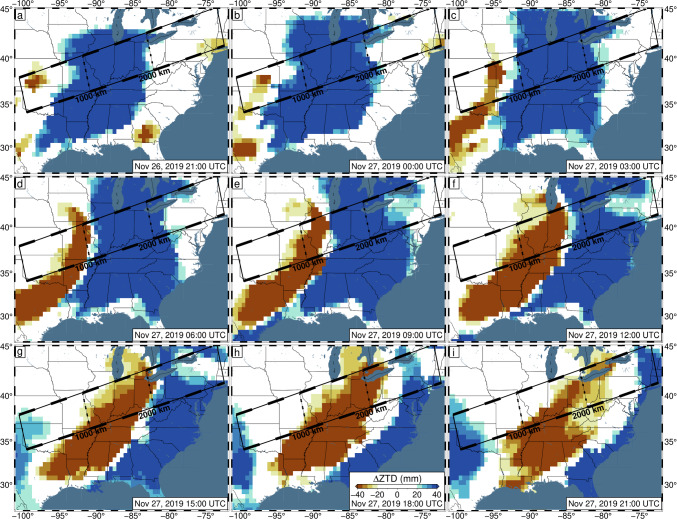
Same as Fig. 9 except for the TROPx08 solution [24 mm/√(hr)]

Most of the storm-induced variation can be accounted for with the TROPx04 solution; however, to fully remove the spatial signal, the TROPx08 solution is required (Fig. 6). At this level, vertical displacements are no longer spatially correlated with the observed radar reflectivity and are now better correlated with deviations in the ZTD estimate and IWV levels (Fig. 7). We find that where systematic displacements were previously observed in the vertical displacements along transect (Fig. 2), no consistent trends are observed at the TROPx08 level (Fig. 8). The ZTD and IWV estimates reveal the opposite, with the TROPx08 solution exhibiting increased variability at the times where large vertical displacements were estimated by the TROPx01 solution (Figs. 9, 10, S1, S2, and S3). A comparison of the TROPx01 and 08 solutions can be viewed in Videos S1, S2, and S3, which provide a time lapse of vertical displacement, ZTD deviation, and water vapor for each solution on November 27, 2019. We find that throughout the TROPx08 time lapse, ZTD values reach peak deviations earlier than with the TROPx01 solution and the IWV gradient between air masses is much more rapid (i.e., spread over tens of kilometers rather than a few hundred kilometers during the peak of the storm). Sporadic vertical displacement outliers are not significantly different between the two solutions.

These findings reveal that 5-min GPS repeatability can be substantially improved on stormy days by loosening the random walk constraint. The choice of this value, however, is dependent on the complexity of the atmosphere on the day in question and varies from station to station.

### Regional data quality

**Fig. 11:**
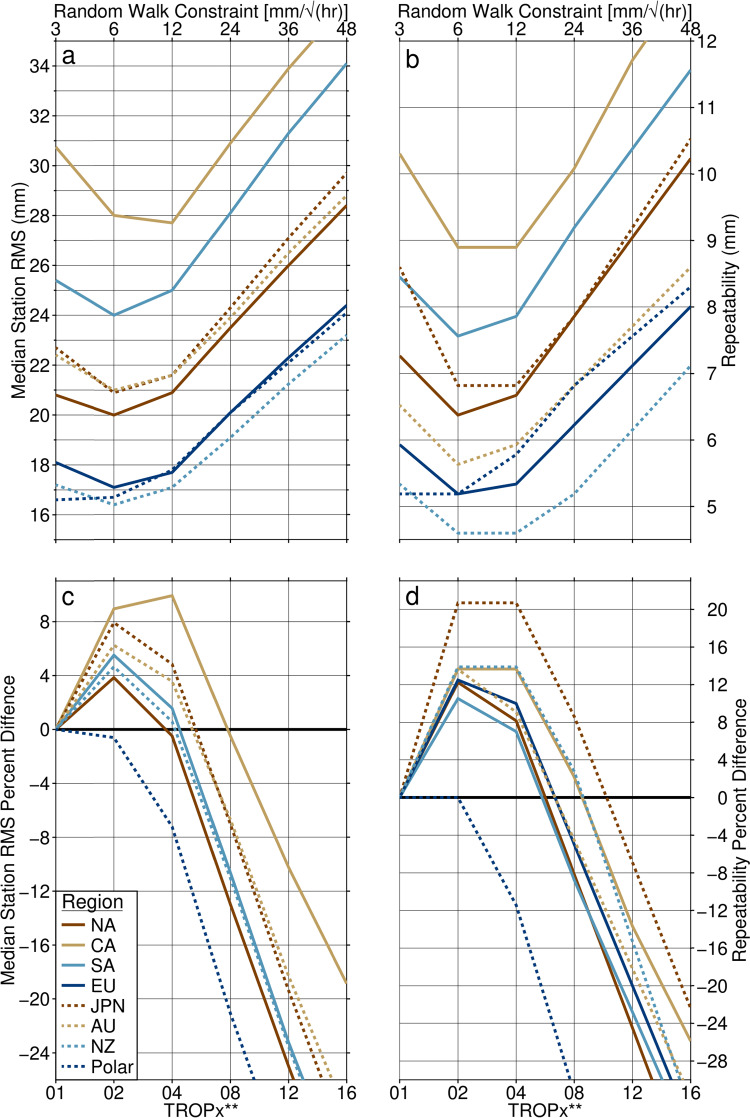
Global 2021 data set comparison by region and processing strategy of the **a** median station vertical RMS, **b** its repeatability, **c** the percent difference of the median station vertical RMS relative to the TROPx01 solution, and **d** its repeatability relative to the TROPx01 solution. Horizontal black line represents equivalence to the TROPx01 solution. Regions are as follows: *NA* North America, *CA* Central America, *SA* South America, *EU* Europe, *JPN* Japan, *AU* Australia, *NZ* New Zealand, and Polar represents stations at latitudes >|± 60°|. Note that all except the polar region, exhibit improvements in both RMS and repeatability in the 6–12 mm/√(hr) range. Values are presented in Table S1

To investigate the effect on the global GPS network of altering the random walk constraint, we first consider its regional impact. Figure 11 shows a comparison between median station 5-min vertical RMS and repeatability of the 2021 data for several regions around the globe. Substantial variation is present in the median station RMS, with the lowest values when using the default random walk, produced by stations in New Zealand and the polar region (which we define as stations located at a latitude >|± 60°|), at 17.2 mm and 16.6 mm, respectively (Table 4). The highest median RMS values are found in Central and South America, with values of 30.8 mm and 25.4 mm, respectively. A similarly large range is observed in station repeatability, with regions exhibiting scatter between 5.2 mm and 10.3 mm. We find that all regions, except for the polar regions, show improvements by loosening the random walk constraint, with both the TROPx02 and 04 strategies performing better than the TROPx01 solution. TROPx02 is the optimal strategy for most regions; however, with respect to repeatability, Central America, Japan, and New Zealand show equivalent improvements with both the TROPx02 and 04 solutions. Improvements in median RMS range between 4 and 9% with the TROPx02 solution, and Central America improves by 10% with the TROPx04 solution. We find that for station repeatability, most regions improve by 10%–14% with the TROPx02 solution. Two exceptions are the polar region, which shows no change, and Japan which improves by 21%. Regional median RMS and repeatability are significantly reduced at the TROPx12 and 16 levels.

**Table 4:** Median station 5-min vertical RMS and repeatability by region of the global 2021 data sets, for the TROPx01, CS, and OP processing strategies

	Median RMS ± Repeatability (mm)		
Region	TROPx01	TROPxCS	TROPxOP
NA	20.8 ± 7.3	20.2 ± 6.7	19.0 ± 5.5
CA	30.8 ± 10.3	27.1 ± 8.5	25.8 ± 7.6
SA	25.4 ± 8.5	24.0 ± 7.6	23.0 ± 7.0
EU	18.1 ± 5.9	17.3 ± 5.3	16.3 ± 4.6
JPN	22.7 ± 8.6	21.1 ± 7.1	19.8 ± 6.1
AU	22.4 ± 6.5	21.1 ± 5.8	20.1 ± 5.0
NZ	17.2 ± 5.3	16.8 ± 5.0	15.7 ± 4.0
Polar	16.6 ± 5.2	16.5 ± 5.0	15.9 ± 4.6
Global	20.7 ± 7.4	19.9 ± 6.7	18.7 ± 5.6

When considering the variable random walk data sets, both are found to produce improved median station RMS and repeatability values relative to TROPx01, with the TROPxOP solution showing substantial improvements (Table 5). Globally, median RMS and repeatability are improved by 4% and 10% with the TROPxCS strategy. For the TROPxOP data set, these improvements increase to 10% and 24%, and station repeatability in Japan improves by 29%. These results reveal that the global GPS network is particularly sensitive to the level of the applied random walk constraint and data quality can be improved by loosening the uniform value or by applying a variable strategy.

**Table 5:** Median station 5-min vertical RMS and repeatability percent difference relative to the TROPx01 solution for the TROPxCS and OP results presented in Table 4

	TROPxCS		TROPxOP	
Region	Median RMS percent difference	Repeatability percent difference	Median RMS percent difference	Repeatability percent difference
NA	3	8	9	24
CA	12	18	16	26
SA	6	11	9	18
EU	4	10	10	23
JPN	7	17	13	29
AU	6	11	10	23
NZ	2	6	9	25
Polar	1	3	4	11
Global	4	10	10	24

### Regional variation

**Fig. 12:**
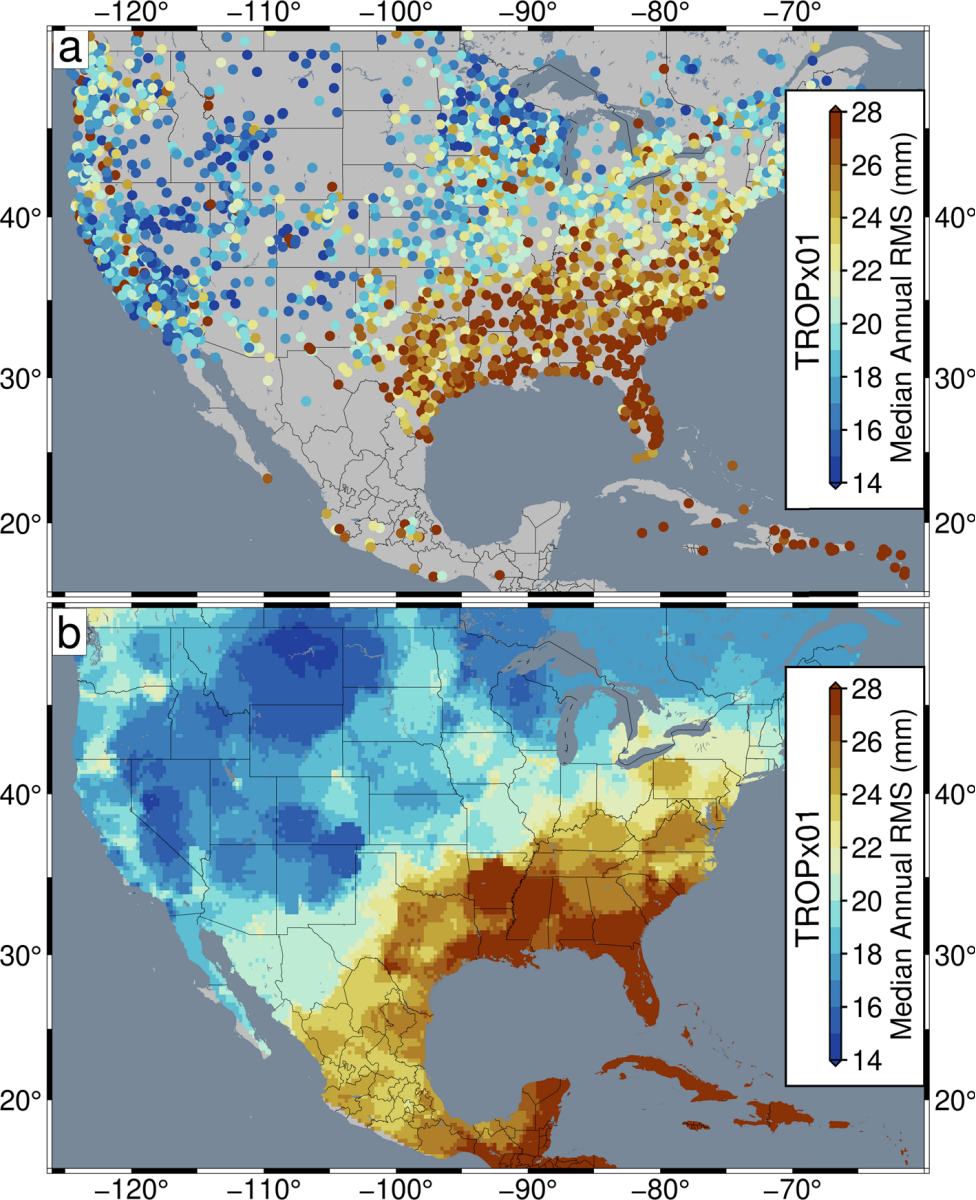
TROPx01 strategy **a** median annual vertical RMS; **b** median annual vertical RMS smoothed by Robust Network Imaging, for GPS stations in the USA, Central America, and the Caribbean

Closer inspection of the regional distribution of 5-min vertical RMS values, reveals substantial variation dependent on the local climate. Areas where continental/maritime tropical air masses are typically present exhibit elevated annual RMS values, while locations where maritime/continental polar air masses are expected, tend to exhibit the lowest RMS values. In North America, this is dramatically present, with much of the southeastern USA exhibiting RMS values > 24 mm, while the western and central states exhibit RMS values under 20 mm (Fig. 12). The sharp gradient between low and elevated RMS values aligns well with the interaction of dry southeast-bound continental polar air masses, with moist northeast-bound tropical air masses from Mexico and the Gulf of Mexico (Aguado and Burt 2013). Similar distributions are observed between the Mediterranean region and northern Europe (Fig S4), as well as between southern and northern Japan (Fig S5), with areas of consistent maritime/tropical air masses exhibiting elevated station RMS.

**Fig. 13:**
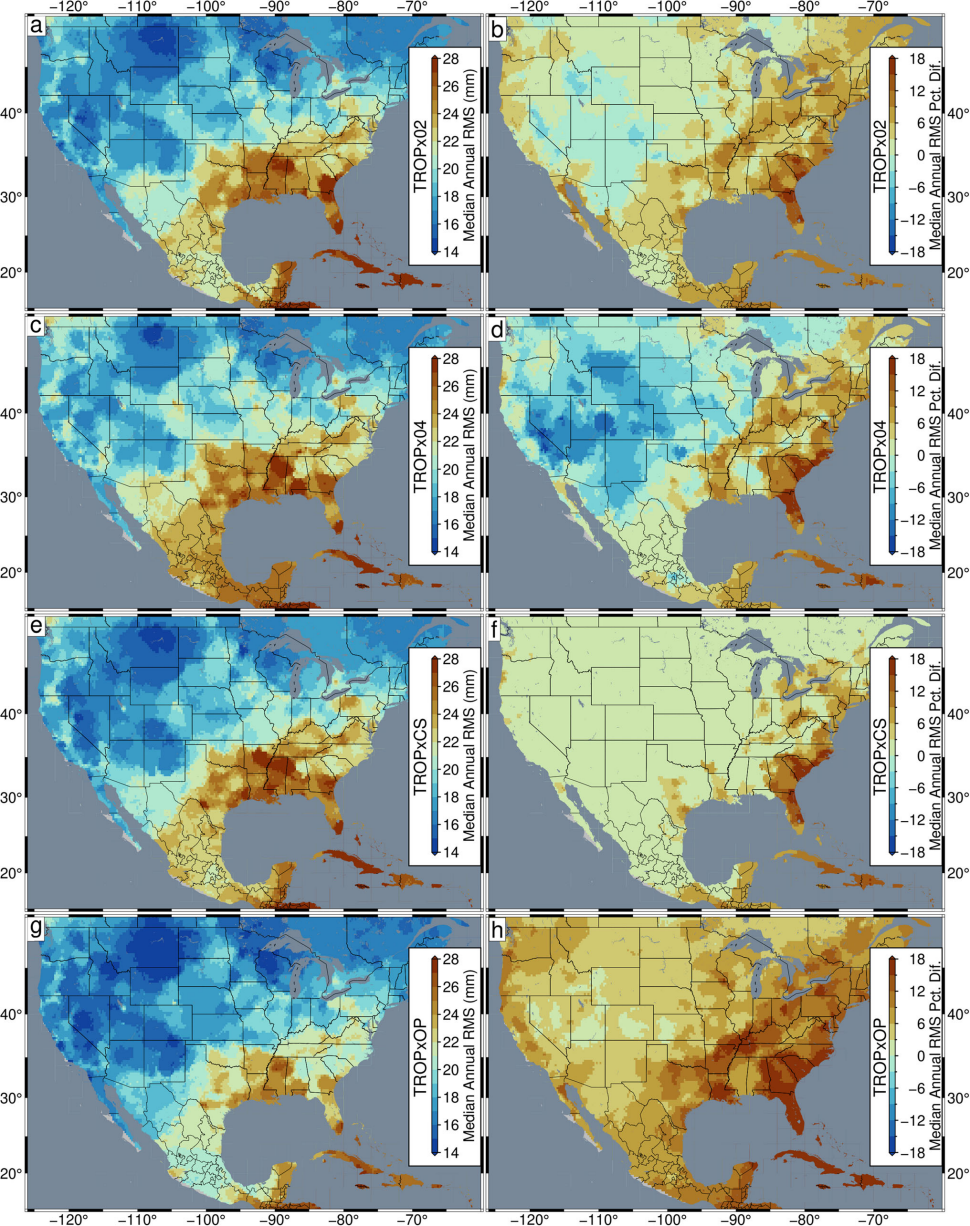
Comparison of **a**, **c**, **e**, and **g** Robust Network Imaged median annual vertical RMS and its **b**, **d**, **f**, and **h** percent difference relative to the TROPx01 solution for the (**a** and **b**) TROPx02, **c** and **d** TROPx04, **e** and **f** TROPxCS, and **g** and **h** TROPxOP processing strategies for GPS stations in the USA, Central America, and the Caribbean

Figure 13 shows distributions of Robust Network Imaged median annual vertical RMS (Kreemer et al. 2020), and its percent difference relative to the TROPx01 solution, for the USA, Central America, and the Caribbean, for the TROPx02, 04, CS, and OP strategies. Results are limited to the 2021 data, from which the median of the RMS of all calculated epochs of the year, for each station, is spatially filtered onto a 0.25° grid. The TROPx02 solution shows that most regions exhibit improvements, except for the Intermountain West which exhibit reductions of less than 3%. The largest improvements are observed in the southeastern USA and the Caribbean. At the TROPx04 level, most of the western and central USA see reductions in data quality of up to 12%; however, Florida, Georgia, South Carolina, and the Caribbean see larger improvements than the TROPx02 solution.

**Fig. 14:**
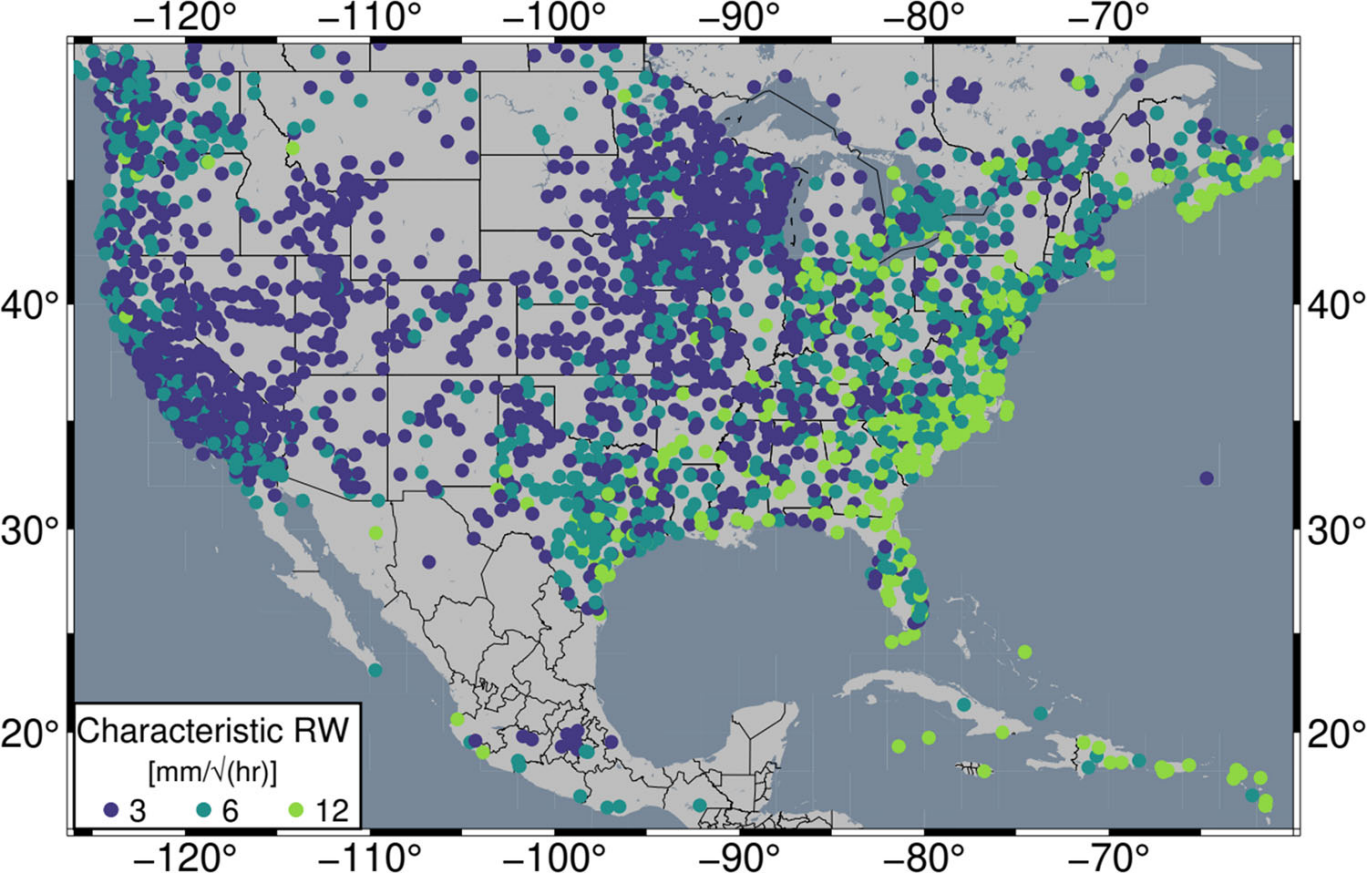
Characteristic station random walk constraint for GPS stations in the USA, Central America, and the Caribbean. Values reflect the processing strategy which most frequently minimizes station RMS in 2021

When considering the spatially variable TROPxCS strategy, all regions are improved or equivalent to the TROPx01 solution, but the majority is improved by less than three percent. Only the eastern seaboard and the Caribbean exhibit improvements greater than nine percent. The attributed characteristic random walk values, per station, are shown in Fig. 14. Most of the continental interior tends toward the default random walk as optimal, while RMS values in the eastern USA are most frequently minimized at random walk values of 6 and 12 mm/√(hr). In both Europe and Japan, most stations exhibit a characteristic random walk higher than 3 mm/√(hr) (Table 6, Figures S6 and S7). Globally, 47.7% of stations retain 3 mm/√(hr) as their characteristic random walk constraint.

**Table 6:** Percentage of stations per characteristic random walk constraint, by region. Values indicate the percentage of stations which most frequently exhibit the lowest station RMS per strategy. Distributions are plotted in Figs. 14, S6, and S7. Globally, only three stations are characteristic at 24 mm/√(hr)

	Strategy ID			
	01	02	04	
Region	Percentage of stations			Station count
NA	56.5	31.8	11.7	3003
EU	42.6	50.3	6.9	923
JPN	27.6	54.0	18.4	976
Global	47.7	40.4	11.8	5819

The largest improvements are observed with the daily optimal strategy. Here, all regions exhibit improvements, with most regions greater than 6%, and much of the southeastern USA and the Caribbean improves by more than 18%. Similar levels of improvements are observed with each processing strategy in Europe (Fig. S8) and Japan (Fig. S9), with Japan showing significant improvements with the TROPxOP strategy, at 29%.

### Annual variability

**Fig. 15:**
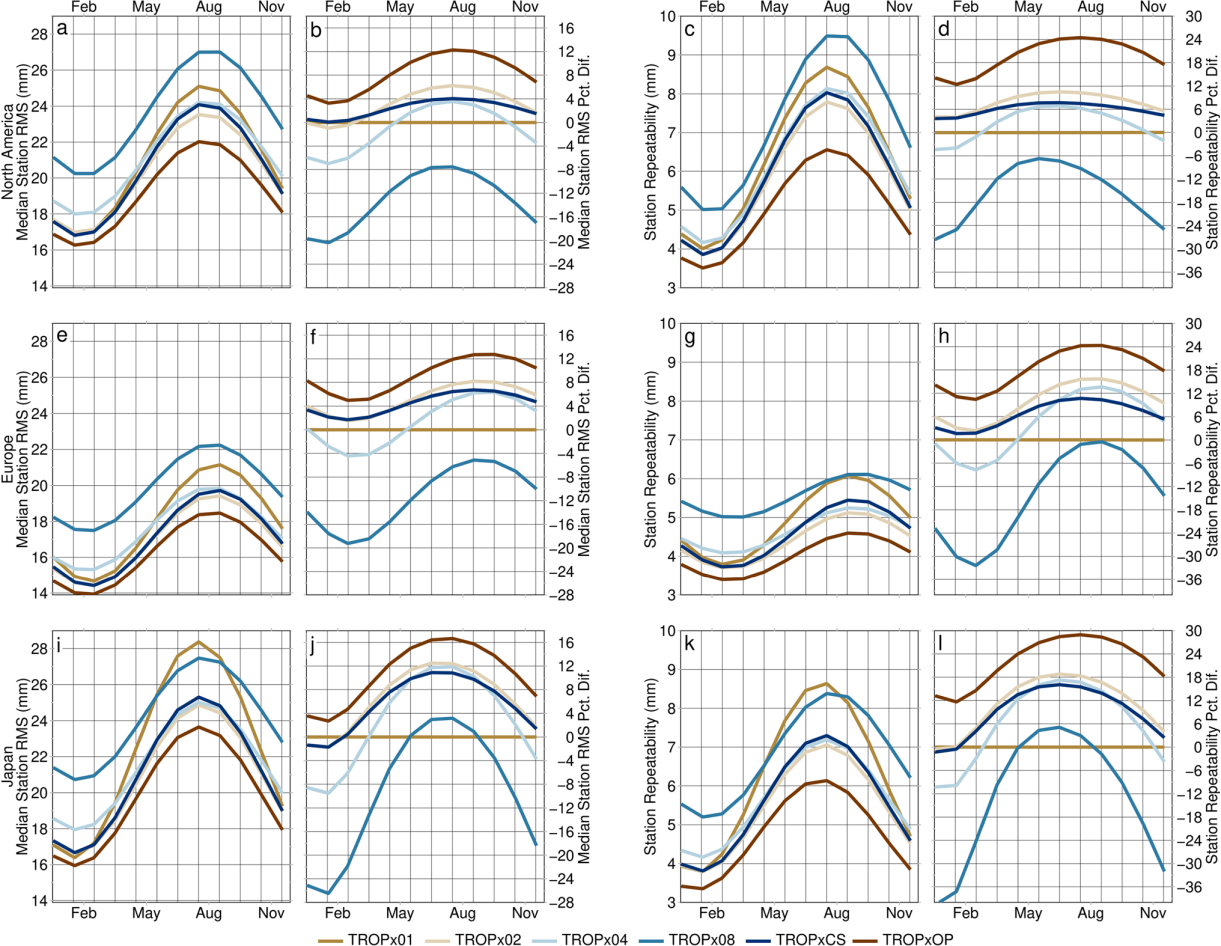
Annual component by processing strategy of (**a**, **e**, and **i**) the fit to the median station 5-min vertical RMS, **b**, **f**, and **j** its percent difference relative to the TROPx01 solution, **c**, **g**, and **k** the fit to the station vertical repeatability, and **d**, **h**, and **l** its percent difference relative to the TROPx01 solution, for **a**–**d** North America, **e**–**h** Europe, and **i**–**l** Japan

**Fig. 16:**
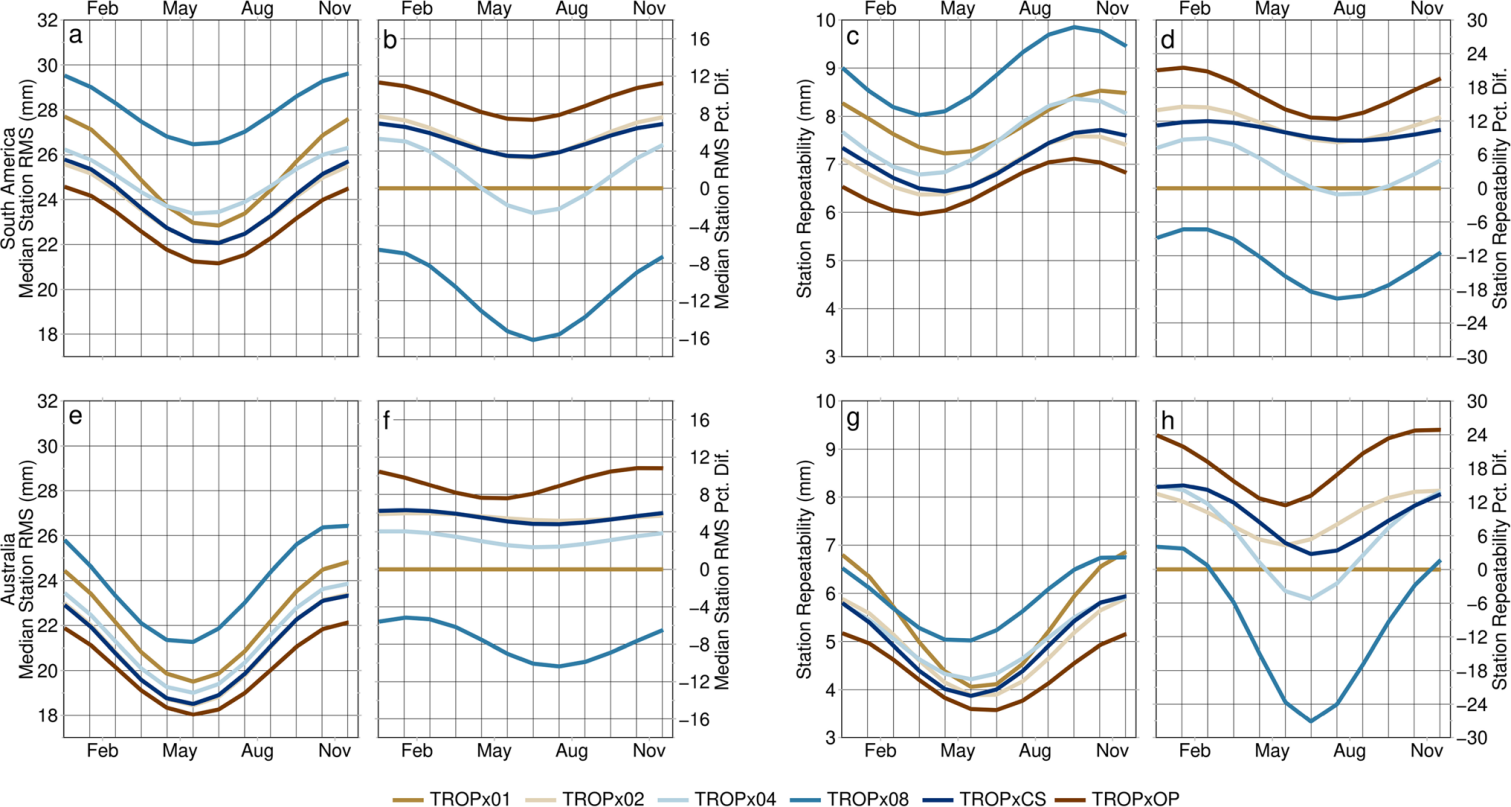
Annual component by processing strategy of **a** and **e** the fit to the median station 5-min vertical RMS, **b** and **f** its percent difference relative to the TROPx01 solution, **c** and **g** the fit to the station vertical repeatability, and **d** and **h** its percent difference relative to the TROPx01 solution, for **a**–**d** South America and **e**–**h** Australia. Note that for median station vertical RMS, the TROPx02 and TROPxCS solutions are nearly identical

A strong annual signal is present in both the station 5-min vertical RMS and its repeatability, and substantial variation is present between regions. As expected, the annual signal is of opposite sign between northern hemisphere (Fig. 15) and southern hemisphere regions (Fig. 16). These figures reflect the annual component of the fit to daily median 5-min vertical RMS and repeatability values, for each region, on the days processed in 2021. Results for each strategy are then compared to the TROPx01 solution. Peak amplitudes are found during the summer months, when the troposphere is thickest (Rieckh et al. 2014). We find that for all cases, the TROPx02 solution performs equivalent to, or better than, the TROPx01 solution. The TROPx04 solution performs better than current processing during the summer months, by hemisphere, but under-performs during winter, and the TROPx08 solution rarely performs better than the TROPx01 solution. The TROPxCS strategy always performs better than the TROPx01 solution; however, it typically under-performs during the summer months when compared to the TROPx02 solution. The largest improvements to both RMS and repeatability are found in the summer months. Peak improvements are obtained when using the TROPxOP strategy which, in terms of median RMS, are 12%, 13%, 16%, 11%, and 11%, respectively, for North America, Europe, Japan, South America, and Australia. For station repeatability, peak improvements are 24%, 24%, 29%, 22%, and 25%, respectively, each of which are roughly twice the level of improvement achieved by the TROPx02 and TROPxCS strategies. These results highlight significant annual variability in data quality, dependent on the choice of the random walk constraint.

### Daily position improvements

**Fig. 17:**
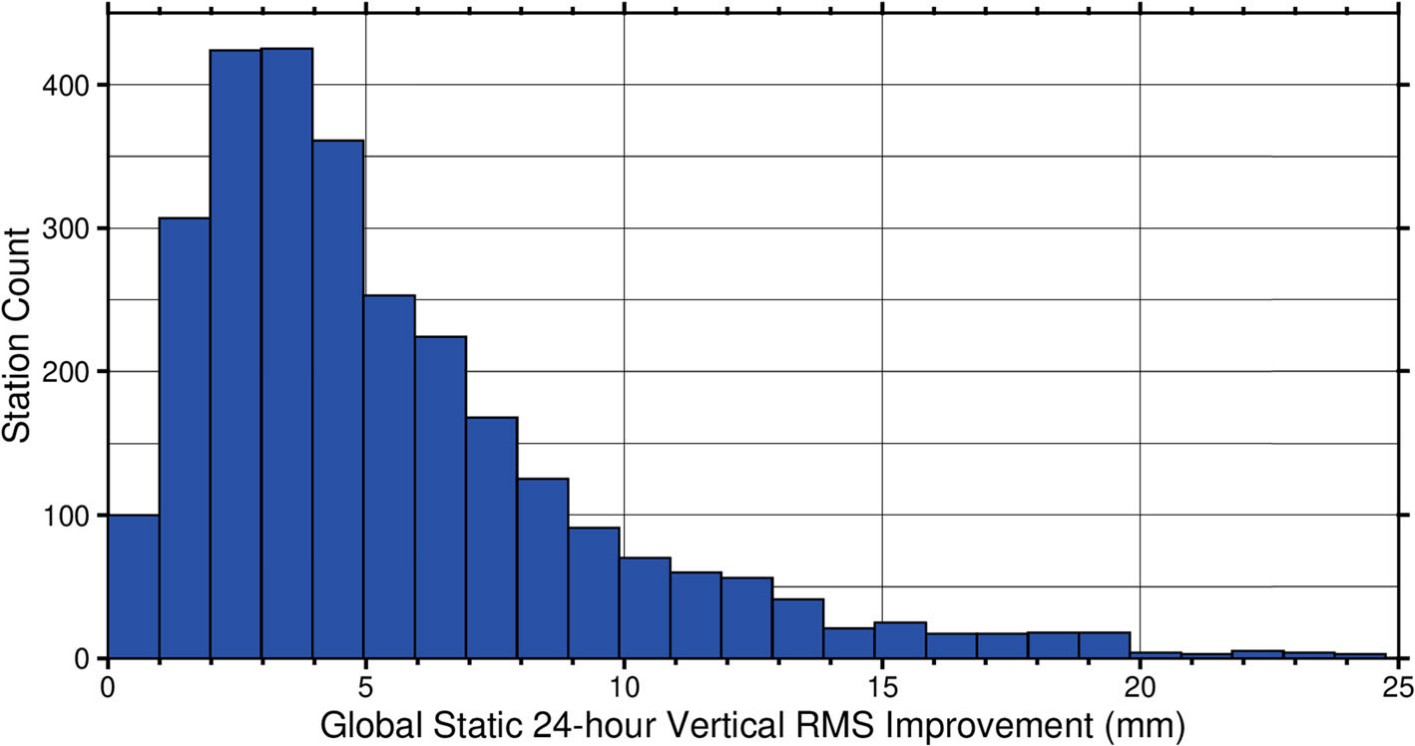
Global static 24-h position vertical annual RMS improvement for the 2021 data set. Values are defined following √(RMS1^2^-RMS0^2^). Here, RMS1 represents the station RMS values of the TROPx01 solution of the 24-h static positions for the year, and RMS0 represents the value which best minimizes the station RMS by strategy. Note that solutions which maintain the TROPx01 solution as optimal [1356 stations (32%)], and thus show no difference, are not plotted here

Inspection of the static 24-h positions of our 2021 data set reveals that most stations exhibit improvements at looser random walk values than with the default constraint. To determine this, we calculate the annual RMS improvement for each station. This is defined as √(RMS1^2^-RMS0^2^), where RMS1 represents the station RMS values of the TROPx01 solution, and RMS0 represents the value which best minimizes the station RMS by strategy. Prior to calculating the annual RMS values, the linear trend for each station is removed; however, the annual signal is retained. We find that 32% of stations retain the TROPx01 strategy, while the rest of the stations exhibit improvements (Fig. 17). The median improvement for the global daily positions is 4.5 ± 2.8 mm. These results indicate that the improvements exhibited at the 5-min processing level by loosening the random walk constraint, propagate into the static 24-h positions.

## Discussion

### Weather and sub-daily positioning

We present clear evidence that the vertical displacements observed by 5-min GPS time series throughout the central/eastern USA on November 26–27, 2019, are artifacts that occur due to rapid atmospheric variation caused by Winter Storm Ezekiel. When using the default random walk constraint of 3 mm/√(hr), ZTD estimates are extremely smooth temporally, regardless of atmospheric variation. This can be seen in Fig. 4, where the TROPx01 solution shows minimal short-term (under two hour) variability on both days, even though atmospheric conditions were significantly more turbulent on November 27th than the 26th. Relaxing the random walk constraint, allows rapid variation between high- and low-pressure fronts to be better accounted for, resulting in substantially improved position time series, and more dynamic ZTD estimates. While large improvements in data quality during the storm are achieved using the TROPx04 and 08 strategies, we also find that on November 26th, both the TROPx02 and 04 strategies reduced vertical station scatter, relative to the TROPx01 strategy. This supports that even on calmer days, loosened constraints improve vertical positioning, and the consistency between the optimal RMS values across both days indicates improved long-term repeatability when the constraints are allowed to vary.

There is a balance between fully accounting for the displacements associated with the storm and reducing the influence of measurement noise. We see the largest data improvements with the TROPxOP solution which produces a much more normal distribution of station vertical RMS and improves repeatability during the storm by 49%. This however does not necessarily indicate that the spurious signal has been corrected fully; rather, it has been suppressed sufficiently such that it does not skew the distribution of the scatter. This suggests that for studies which inspect changes in sub-daily positions (such as early co-seismic displacement fields), a slightly looser constraint is necessary than is required to produce improved daily positions.

Another factor that sees improvement, is the day-to-day coherency for both station position and ZTD. Often, when combining multiple days of sub-daily time series, there is a step in the data at the day transition. This is driven by differences in the models between days, but we find a large portion can be accounted for by allowing the ZTD more freedom (Fig. 4). With a looser random walk constraint, the data are able to overcome discrepancies between the model and observations and are more consistent across the day transition. In the case of station SCCB, this translates to an offset correction of 32 mm between November 26 and 27 when applying the TROPx04 strategy. While we focus on improving positioning, the corrected ZTD estimates result in improved water vapor estimates, presenting an opportunity to improve meteorological studies as well by loosening the random walk constraint.

### Implications of global network positioning improvements

At the global scale, regional and temporal variability in the optimal level of the random walk constraint is substantial for both the uniform and variable approaches. We show that for regions which tend to experience polar air masses (i.e., higher latitudes and most of the western and central USA), the current default value is generally appropriate. However, substantial reduction of station time series scatter is possible for the rest of the global network at values of 6 mm/√(hr) or higher. Coastal regions, where tropical air masses tend to be present, require the loosest random walk constraint and exhibit improvements that are often greater than 15%. A spatially variable random walk constraint approach has not been applied to large network analysis in the past; however, the prevalent heterogeneity of characteristic random walk values shown in Figs. 14, S6, and S7, reveals an excellent opportunity to improve global data repeatability in future reanalysis.

The majority of the results presented in this study focus on improving 5-min positions; however, as shown in Fig. 17, we find that improvements produced by loosening the random walk constraints propagate into the static 24-h positions of the stations. The full impact of loosened constraints on static 24-h positions is the subject of future research; however, clear implications are present toward all aspects of GPS positioning. Reduced scatter in 24-h static positions will produce improved velocity estimates, more accurate co-seismic measurements, and allow for better identification and modeling of temporally variable signals. Additionally, such improvements to the global network will propagate into improved reference frames and satellite orbit determination. Thus, upon identification of revised processing strategies, a global reanalysis will be necessary.

### Recommended processing strategies

In this study, we explored three types of random walk constraint applications, the uniform value, characteristic station specific, and daily variable applications. Using a uniform value is the default approach for GPS processing. We show that while for some regions data quality improves dramatically as the random walk is loosened (i.e., the southeastern USA, the Mediterranean, and southern Japan, see Figs. 13, S8, S11), other areas see large reductions (i.e., the western/central USA). This trade-off may be acceptable for localized studies which produce their own data; however, for global network processing, increasing the random walk constraint > 12 mm/√(hr) uniformly, is not advised. Nevertheless, global data quality is improved by a random walk of 6 mm/√(hr), with only a few regions showing minor reductions, and is our recommended threshold for uniform application.

Applying characteristic station specific (TROPxCS) random walk constraints is of course better than the uniform method, as it allows for regional climatic variability to be accounted for. Additionally, in terms of global network processing, it only requires a simple adjustment to the current processing flow since it would only require defining a suite of values for the network. The characteristic random walk values could then be interpolated to the position of newly installed stations until enough data are present to define the station. With this approach, 52% of stations globally exhibit improvements.

The daily variable (TROPxOP) approach produced superior results compared to the other data sets. This is because it is able to account for not just regional climate variability, but temporal variability associated with both annual fluctuations and passing weather systems. For small-scale studies producing their own data, it is highly recommended to test and identify daily station specific random walk values, regardless of the study area, as we find that even in polar regions (which showed no improvement by uniformly loosening the random walk), repeatability is substantially improved (Table 5). In terms of global network processing, identifying daily optimal random walk constraints using the methods applied here, would scale poorly as the threshold would need to be identified during the processing flow.

An alternative, which would not be as computationally expensive, would be to define an annual random walk per station. As shown in Figs. 15 and 16, annual variation is significant, and peak improvements are obtained during the summer by looser solutions, often with the TROPx04 strategy performing better than the TROPx02 and characteristic strategies. Thus, a time variable random walk per station could range from the TROPx01 strategy, for winter months and the TROPx04 strategy for summer, by determining a seasonal amplitude and phase term per station. We leave determination of an efficient identification method to future studies, but note that for the 2021 data set, 63.8% of station days exhibited RMS reductions by allowing the random walk to vary each day, per station.

## Conclusions

In this study, we present a sensitivity analysis of the ZTD random walk constraint on 5-min GPS positions at local and global scales. Given that the RMS variation of weighted mean 5-min positions closely replicates that of 24-h static solutions to ~ 0.1 mm (Blewitt 2015), our ultimate goal is to improve 24-h static positions and other 24-h batch products, such as precise orbit parameters.

We show that station vertical RMS and repeatability can be improved by loosening the random walk constraint from the GipsyX default value of 3 mm/√(hr) to 6–24 mm/√(hr). The choice of the optimal threshold, however, is strongly dependent on the atmospheric, regional, temporal, and climatic conditions of the station. We find that during Winter Storm Ezekiel, applying a random walk constraint of 24 mm/√(hr) more adequately accounts for variations in the ZTD, improving station repeatability by 45%. Optimizing the random walk for each station further improves repeatability to 49% and additionally improves the RMS IQR and IPR by 48% and 43%, respectively.

In the global analysis, under more typical atmospheric conditions, we find that there is substantial opportunity to improve data quality by loosening the random walk. Median vertical RMS and station repeatability are most improved when using a random walk of 6–12 mm/√(hr), and (with the exception of polar stations which do not typically improve), improved by 4–9% and 10–21%, respectively. Additionally, we show that that the improvements observed in the 5-min kinematic time series, propagate into the static 24-h station vertical position, with a median annual RMS improvement of 4.5 ± 2.8 mm.

Regional heterogeneity in the optimal level of the random walk constraint is substantial, indicating a variable approach is more appropriate. Tests of two variable random walk methods each show significant improvements relative to the default random walk. When defining characteristic random walks for individual stations, we find global repeatability to improve by 10%; alternatively, when applying daily optimal station specific random walks, global station repeatability is improved by 24% and in Japan by 29%. While the daily optimal random walk method is preferred, it is difficult to scale to global network analysis and is the subject of future studies. Defining characteristic random walk values (preferably as an annual sinusoid rather than a uniform value), would be a simple adjustment in the data flow and could be implemented rapidly, resulting in significant improvements in station repeatability for global GPS network processing. At minimum, for uniform application within the GipsyX software, increasing the default value of the random walk constraint to 6 mm/√(hr) is recommended. The random walk constraint values presented in this study reflect 5-min data rates for both kinematic and static processing, and must be scaled by √(*x*/300) for alternative data intervals of *x* seconds.

More general impact on improving GPS positioning may be achieved when implementing our findings in the routine production of precise GPS orbits by analysis centers of the International GNSS Service. To gain maximum benefit, the user of such orbits would then need to adopt the same level of random walk constraint on tropospheric parameters. For software (other than GipsyX) that implements a different method of tropospheric estimation, research would need to be carried out to allow a similar level of tropospheric variation.

## Supplementary Information

Below is the link to the electronic supplementary material.

Supplementary file1 (DOCX 17099 KB)

Supplementary file2 (AVI 26149 KB)

Supplementary file3 (AVI 26052 KB)

Supplementary file4 (AVI 20009 KB)

## Data Availability

GPS time series generated for this study are available from the Nevada Geodetic Laboratory upon request.
