# Supplementary material for: Improved GPS tropospheric path delay estimation using variable random walk process noise
Source: J Geod. 2024 Oct 7;98(10):89. doi: 10.1007/s00190-024-01898-3 (PMC11458690; doi:10.1007/s00190-024-01898-3)
Supplement: Supplementary file 1 — Supplementary file1 (DOCX 17099 KB) [file 190_2024_1898_MOESM1_ESM.docx]

Supplemental material for...

**Improved GPS Tropospheric Path Delay Estimation Using Variable Random Walk Process Noise**

Zachary M. Young^1^, Geoffrey Blewitt^1^, and Corné Kreemer^1^

^1^Nevada Bureau of Mines and Geology, University of Nevada, Reno, NV, USA

This material consists of Table S1, Figures S1 – S9, and Videos S1 – S3

**Supplemental Tables**

**Table S1**: Global median vertical RMS, its repeatability, and their percent difference relative to the TROPx01 solution, by region and processing strategy. Data are plotted in Figure 11.

| **TROPx**** | **Median RMS ± Repeatability (mm)** | **RMS Percent Difference** | **Repeatability Percent Difference** |
| --- | --- | --- | --- |
| **Region - Station Days** | | | |
| **North America – 70,320** | | | |
| 01 | 20.8 ± 7.3 | ~ | ~ |
| 02 | 20.0 ± 6.4 | 4 | 12 |
| 04 | 20.9 ± 6.7 | 0 | 8 |
| 08 | 23.5 ± 7.9 | −13 | −8 |
| 12 | 26.0 ± 9.0 | −25 | −24 |
| 16 | 28.4 ± 10.2 | −37 | −41 |
| **Central America – 1,344** | | | |
| 01 | 30.8 ± 10.3 | ~ | ~ |
| 02 | 28.0 ± 8.9 | 9 | 14 |
| 04 | 27.7 ± 8.9 | 10 | 14 |
| 08 | 30.9 ± 10.1 | 0 | 2 |
| 12 | 33.9 ± 11.7 | −10 | −14 |
| 16 | 36.6 ± 13.0 | −19 | −26 |
| **South America – 4,560** | | | |
| 01 | 25.4 ± 8.5 | ~ | ~ |
| 02 | 24.0 ± 7.6 | 6 | 11 |
| 04 | 25.0 ± 7.9 | 2 | 7 |
| 08 | 28.1 ± 9.2 | −11 | −9 |
| 12 | 31.3 ± 10.4 | −23 | −23 |
| 16 | 34.1 ± 11.6 | −34 | −37 |
| **Europe – 20,688** | | | |
| 01 | 18.1 ± 5.9 | ~ | ~ |
| 02 | 17.1 ± 5.2 | 6 | 13 |
| 04 | 17.7 ± 5.3 | 2 | 10 |
| 08 | 20.1 ± 6.2 | −11 | −5 |
| 12 | 22.3 ± 7.1 | −23 | −20 |
| 16 | 24.4 ± 8.0 | −35 | −35 |
| **Japan – 24,192** | | | |
| 01 | 22.7 ± 8.6 | ~ | ~ |
| 02 | 20.9 ± 6.8 | 8 | 21 |
| 04 | 21.6 ± 6.8 | 5 | 21 |
| 08 | 24.3 ± 7.9 | −7 | 9 |
| 12 | 27.1 ± 9.2 | −19 | −7 |
| 16 | 29.7 ± 10.5 | −31 | −22 |
| **Australia – 5,184** | | | |
| 01 | 22.4 ± 6.5 | ~ | ~ |
| 02 | 21.0 ± 5.6 | 6 | 14 |
| 04 | 21.6 ± 5.9 | 4 | 9 |
| 08 | 23.9 ± 6.8 | −7 | −5 |
| 12 | 26.5 ± 7.7 | −18 | −18 |
| 16 | 28.8 ± 8.6 | −29 | −32 |
| **New Zealand – 3,312** | | | |
| 01 | 17.2 ± 5.3 | ~ | ~ |
| 02 | 16.4 ± 4.6 | 5 | 14 |
| 04 | 17.1 ± 4.6 | 1 | 14 |
| 08 | 19.1 ± 5.2 | −11 | 3 |
| 12 | 21.3 ± 6.2 | −24 | −15 |
| 16 | 23.2 ± 7.1 | −35 | −33 |
| **Polar – 4,656** | | | |
| 01 | 16.6 ± 5.2 | ~ | ~ |
| 02 | 16.7 ± 5.2 | −1 | 0 |
| 04 | 17.8 ± 5.8 | −7 | −11 |
| 08 | 20.1 ± 6.8 | −21 | −31 |
| 12 | 22.1 ± 7.6 | −33 | −46 |
| 16 | 24.1 ± 8.3 | −45 | −60 |
| **Global – 138,656** | | | |
| 01 | 20.7 ± 7.4 | ~ | ~ |
| 02 | 19.7 ± 6.5 | 5 | 12 |
| 04 | 20.5 ± 6.7 | 1 | 10 |
| 08 | 23.1 ± 7.9 | −12 | −6 |
| 12 | 25.7 ± 9.0 | −24 | −22 |
| 16 | 28.0 ± 10.1 | −35 | −36 |

**Supplemental Figures:**


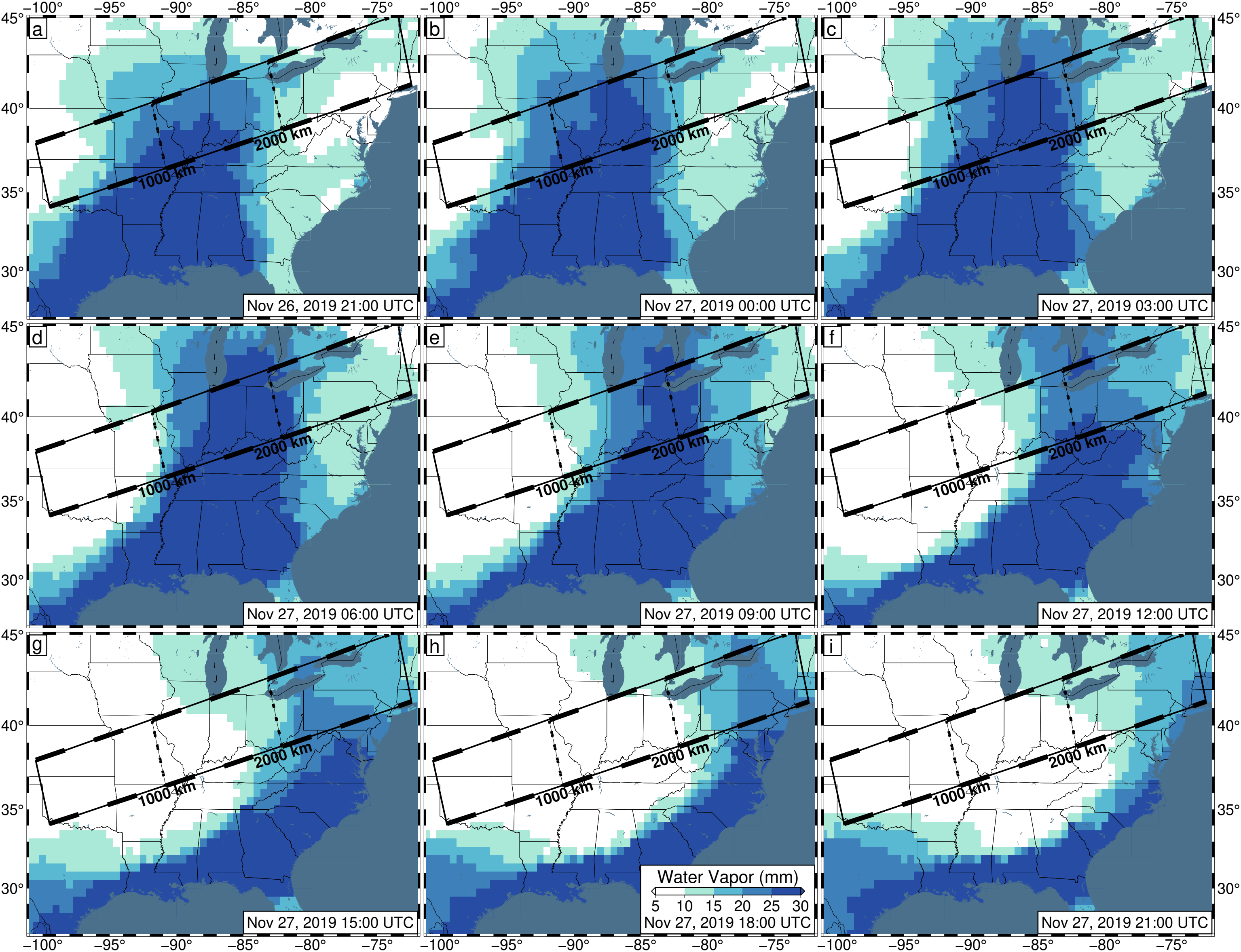


**Figure S1:** Observed integrated water vapor in 3-hour intervals between 21:00 UTC on November 26, 2019, and 21:00 UTC on November 27, 2019. Data have been filtered with Robust Network Imaging and are produced using the current NGL data analysis strategy, with the default ZWD random walk constraint of 3 mm/√(hr).


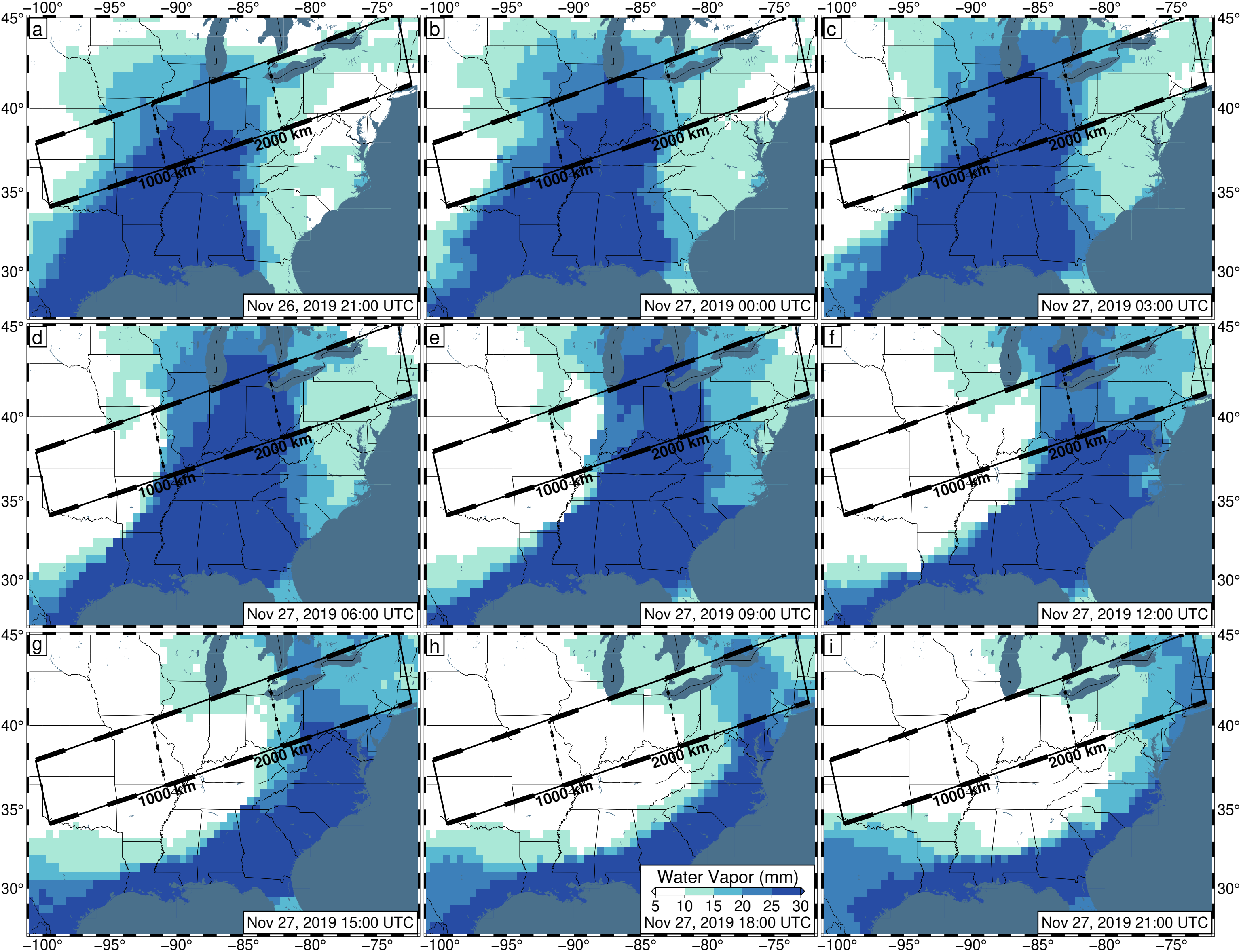


**Figure S2:** Same as Figure S1 except for the TROPx08 solution [24 mm/√(hr)].


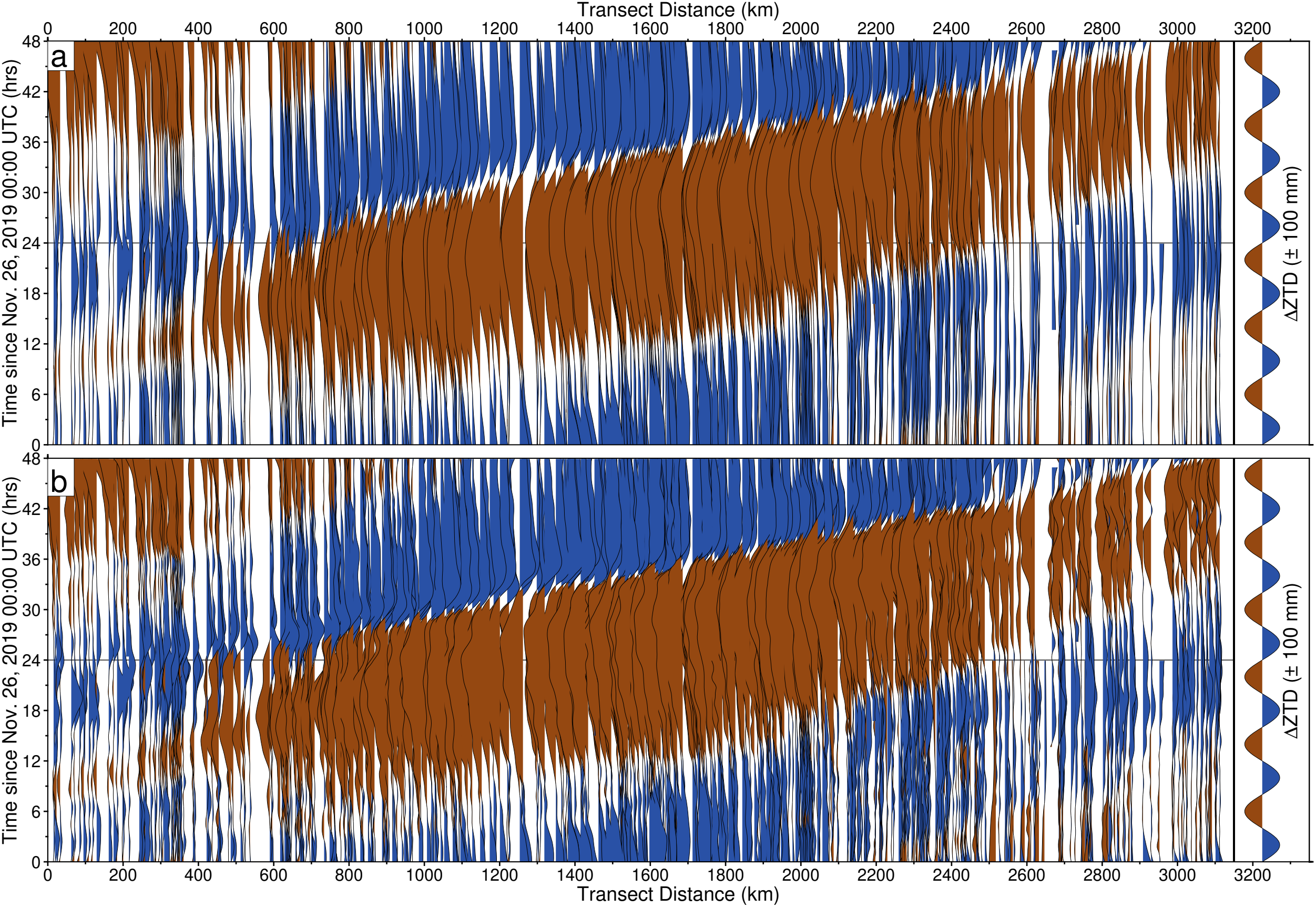


**Figure S3:** Wiggle plots of zenith total delay (ZTD) deviations along the transect identified in Figure 1 for November 26 – 27, 2019, for the **(a)** TROPx01 and **(b)** TROPx08 strategies. For the TROPx08 strategy, deviations are relative to the median station value across November 26 – 27 of the TROPx01 strategy. Data have been smoothed with robust weighted local regression (RLOESS), with a smoothing factor of 0.10. Black horizontal bar represents the start of November 27. Note that the ZTD estimates of the TROPx08 solution exhibit more variability.


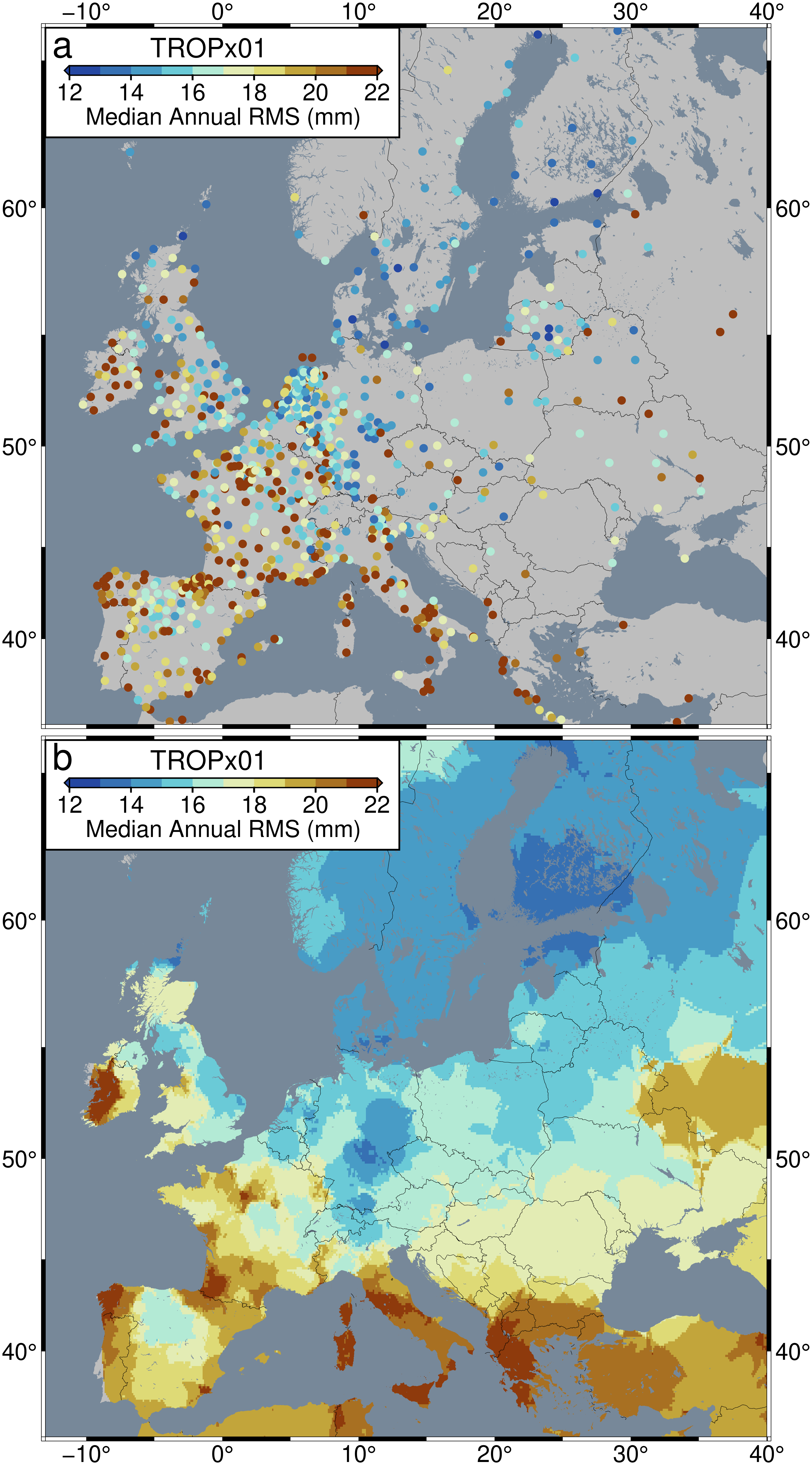


**Figure S4:** TROPx01 strategy **(a)** median annual RMS and **(b)** Robust Network Imaged median annual RMS, for GPS stations across Europe.


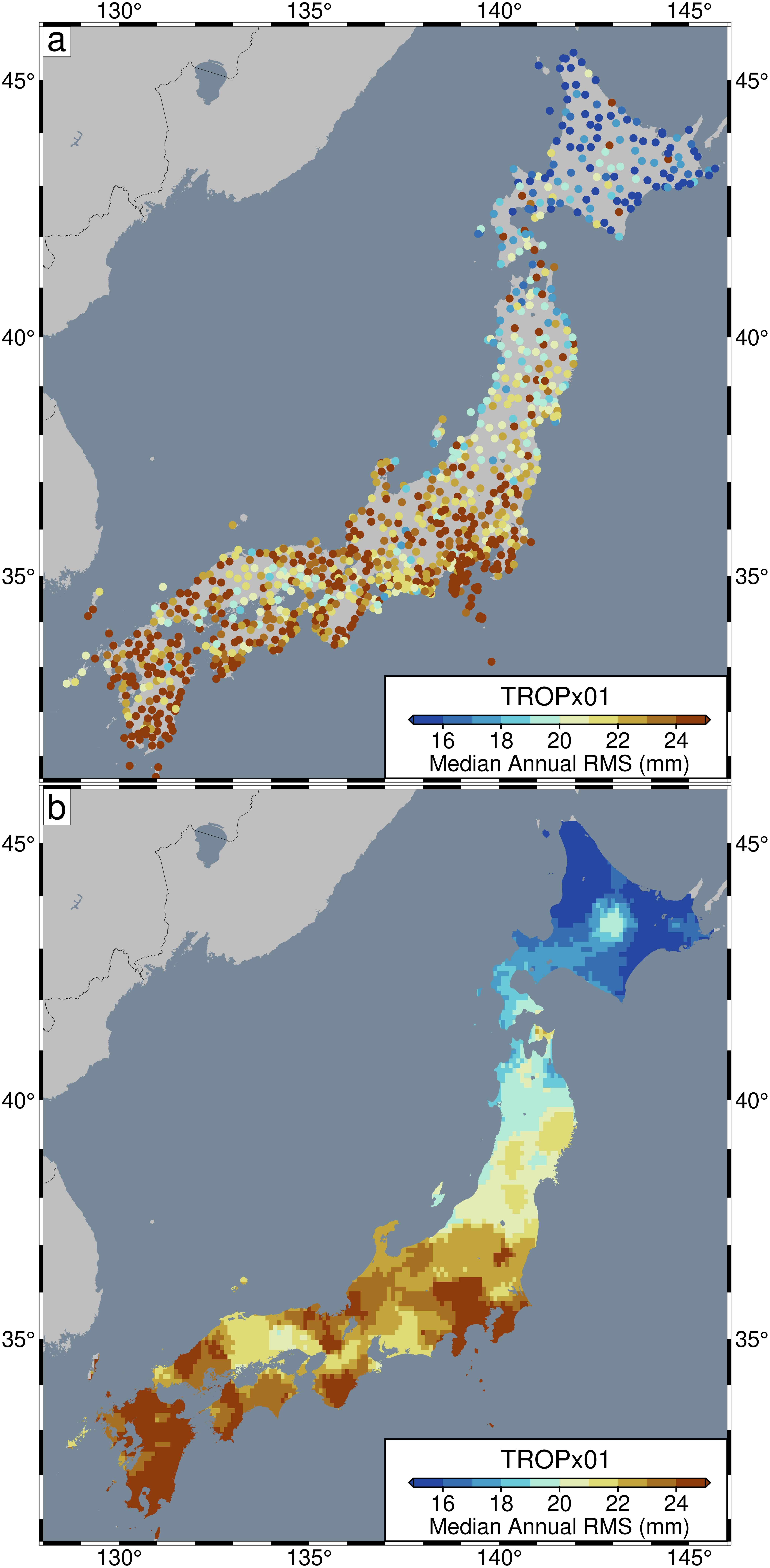


**Figure S5:** TROPx01 strategy **(a)** median annual RMS and **(b)** Robust Network Imaged median annual RMS, for GPS stations in Japan.


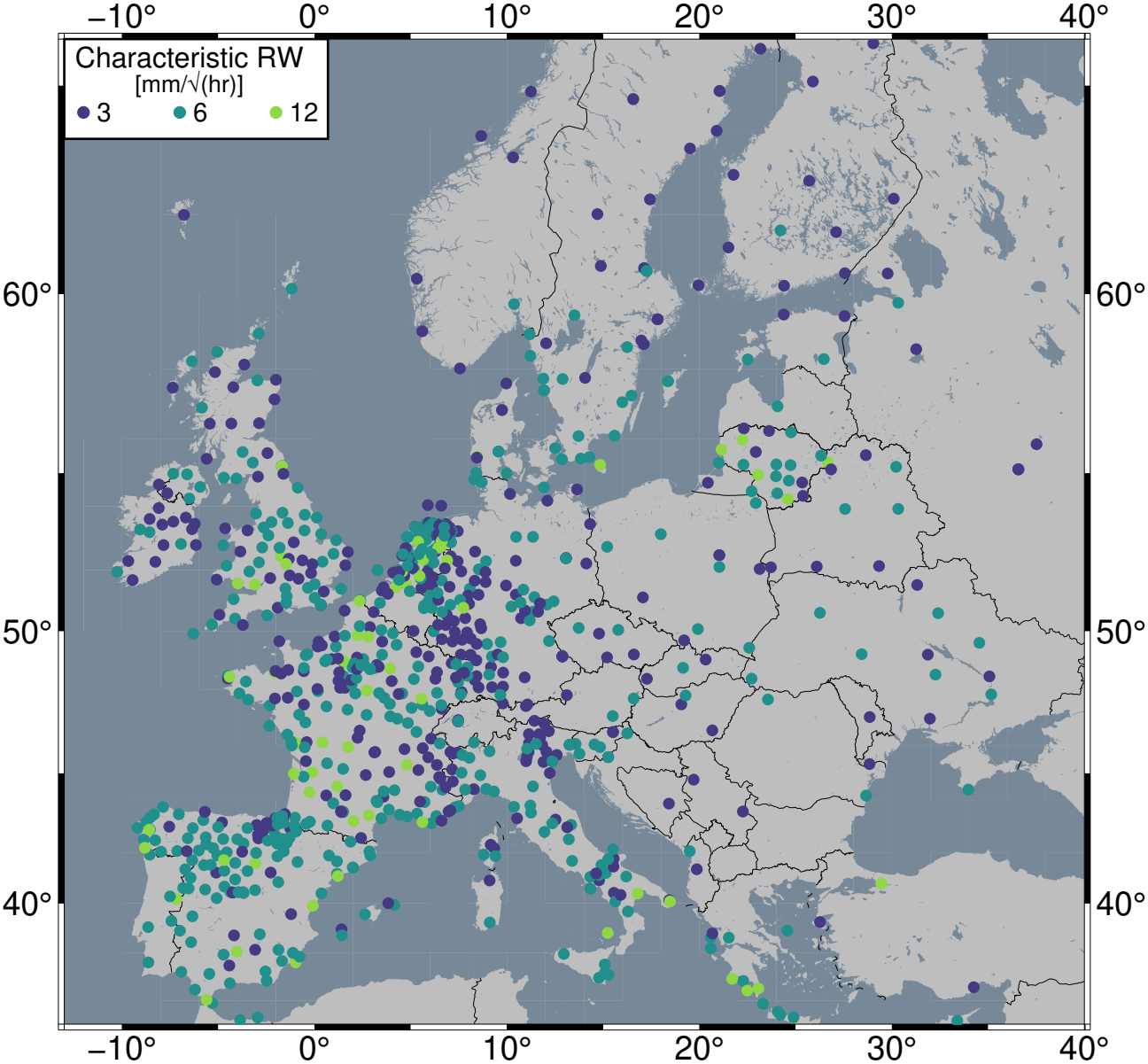


**Figure S6:** Characteristic station random walk constraint for GPS stations across Europe. Values reflect the processing strategy which most frequently minimizes station RMS.


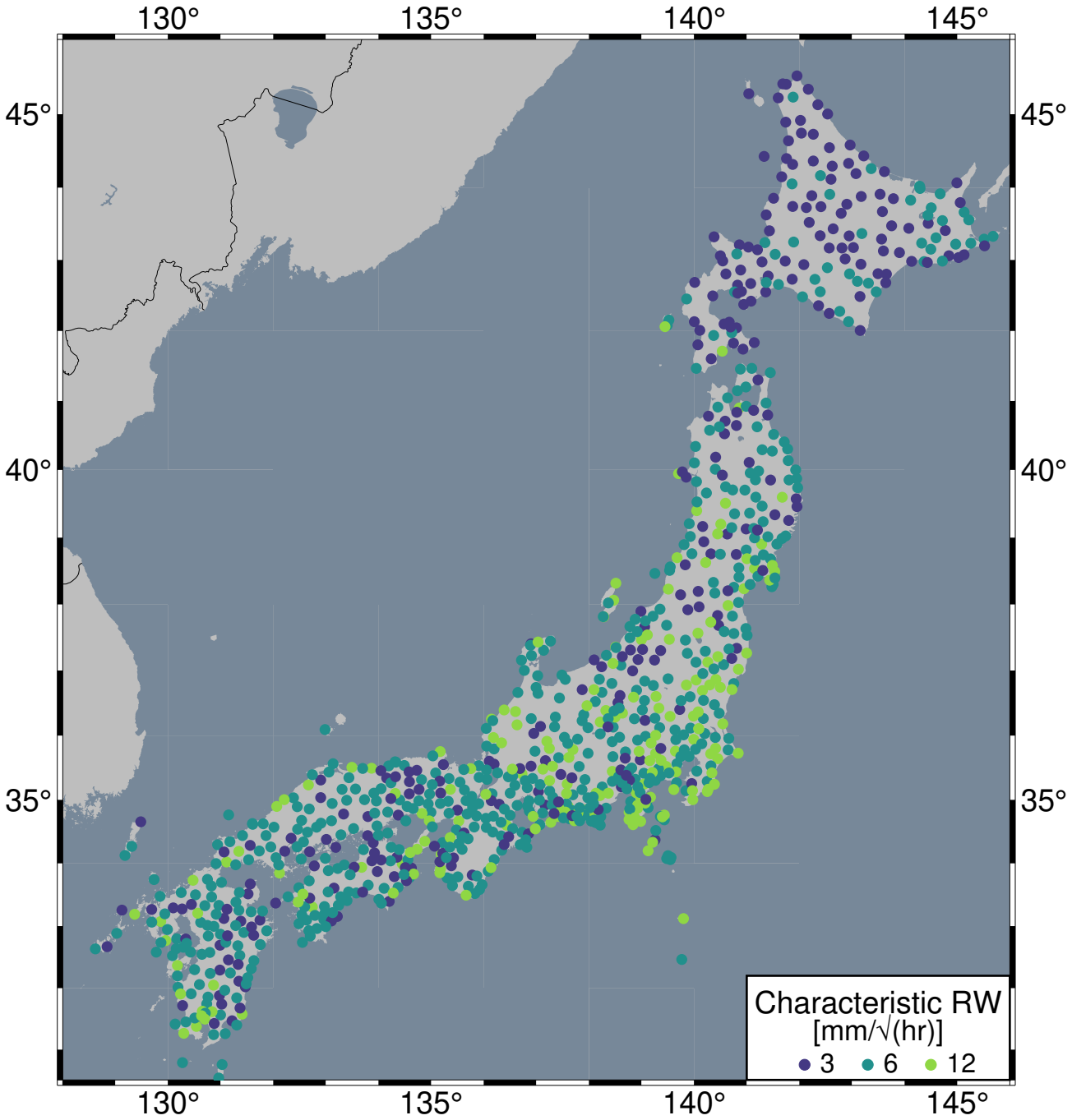


**Figure S7:** Characteristic station random walk constraint for GPS stations in Japan. Values reflect the processing strategy which most frequently minimizes station RMS.


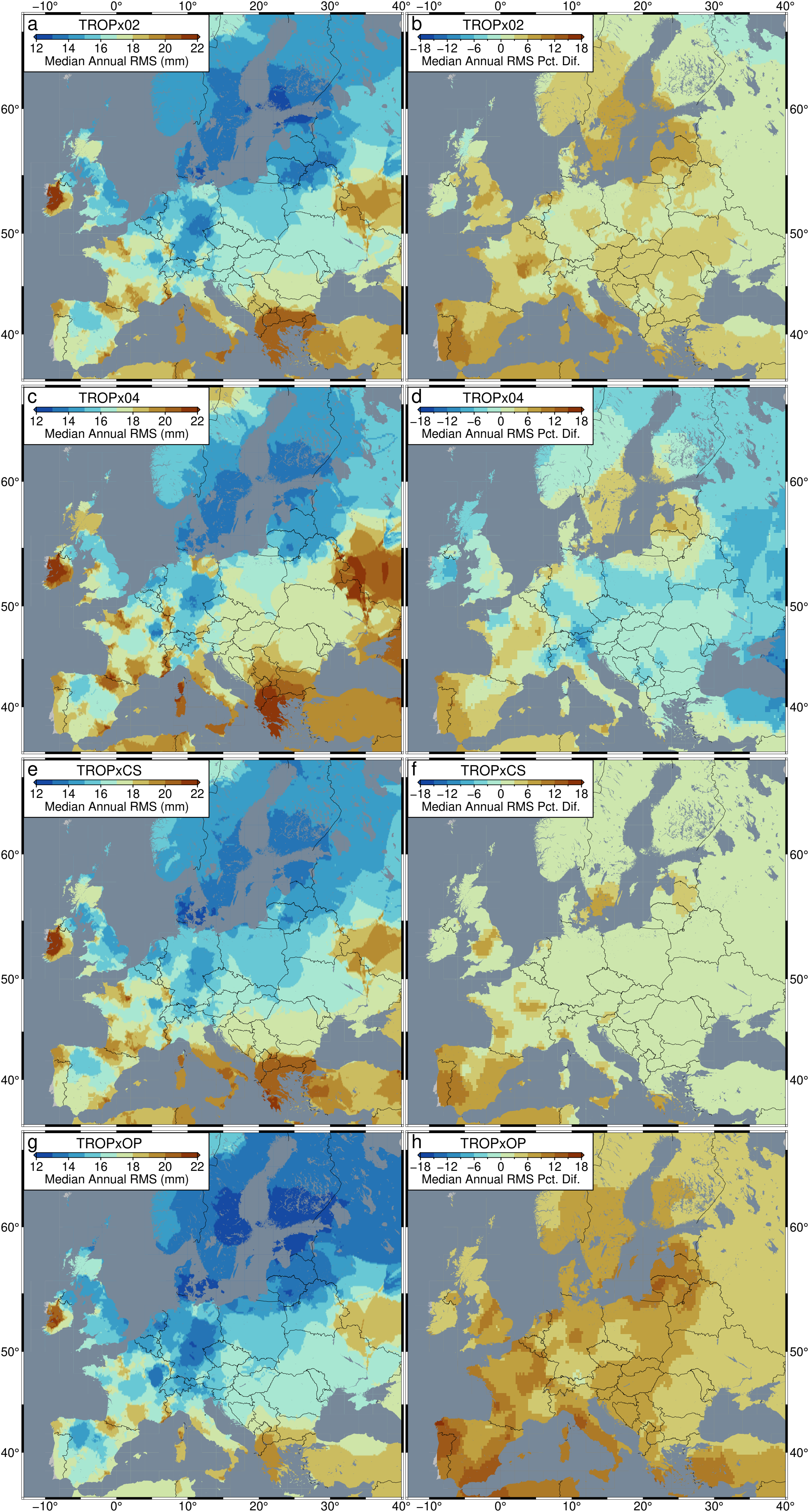


**Figure S8:** Comparison of **(a, c, e, and g)** Robust Network Imaged median annual RMS and its **(b, d, f, and h)** percent difference relative to the TROPx01 solution for the **(a and b)** TROPx02, **(c and d)** TROPx04, **(e and f)** TROPxCS, and **(g and h)** TROPxOP processing strategies for GPS stations across Europe.


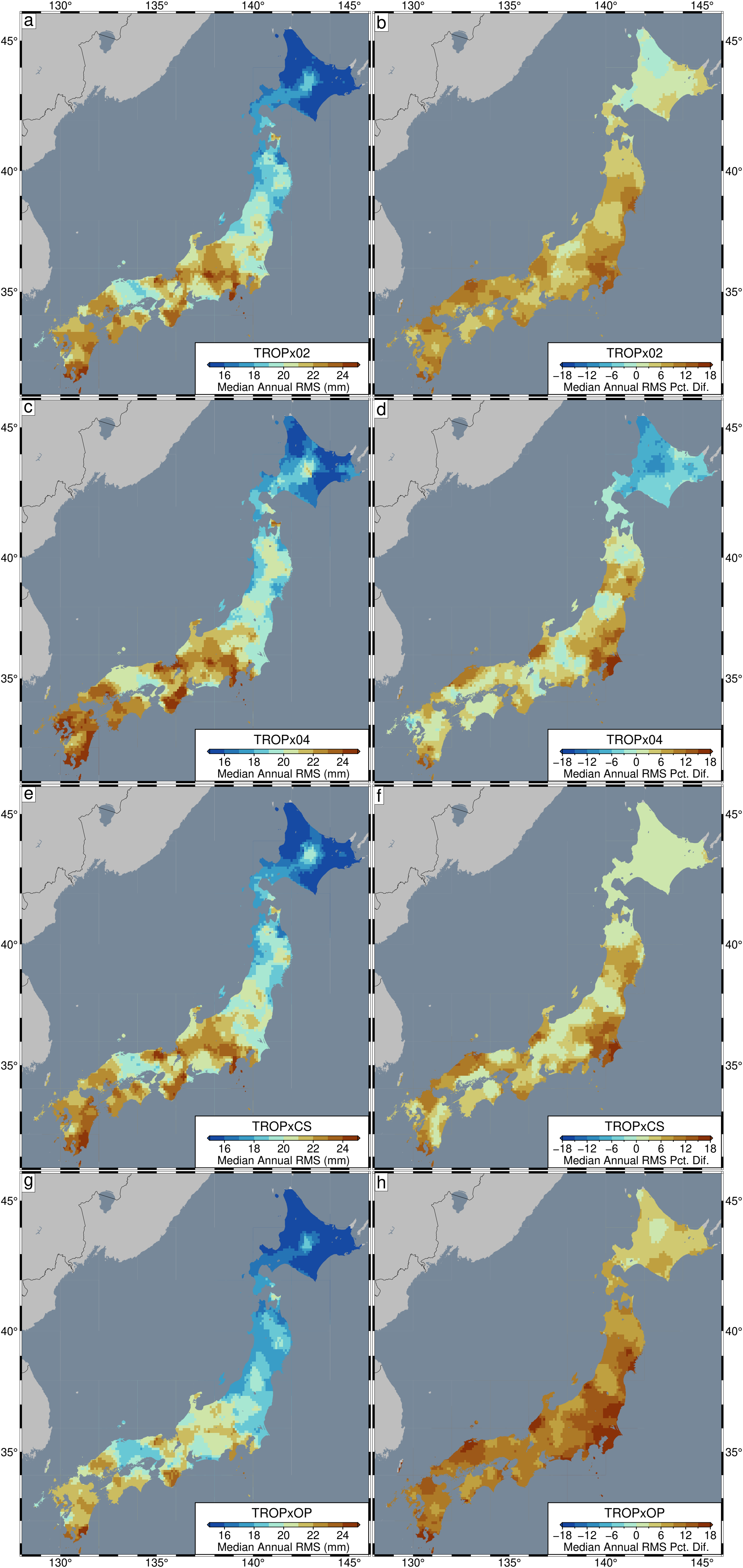


**Figure S9:** Comparison of **(a, c, e, and g)** Robust Network Imaged median annual RMS and its **(b, d, f, and h)** percent difference relative to current processing for the **(a and b)** TROPx02, **(c and d)** TROPx04, **(e and f)** TROPxCS, and **(g and h)** TROPxOP processing strategies for GPS stations in Japan.


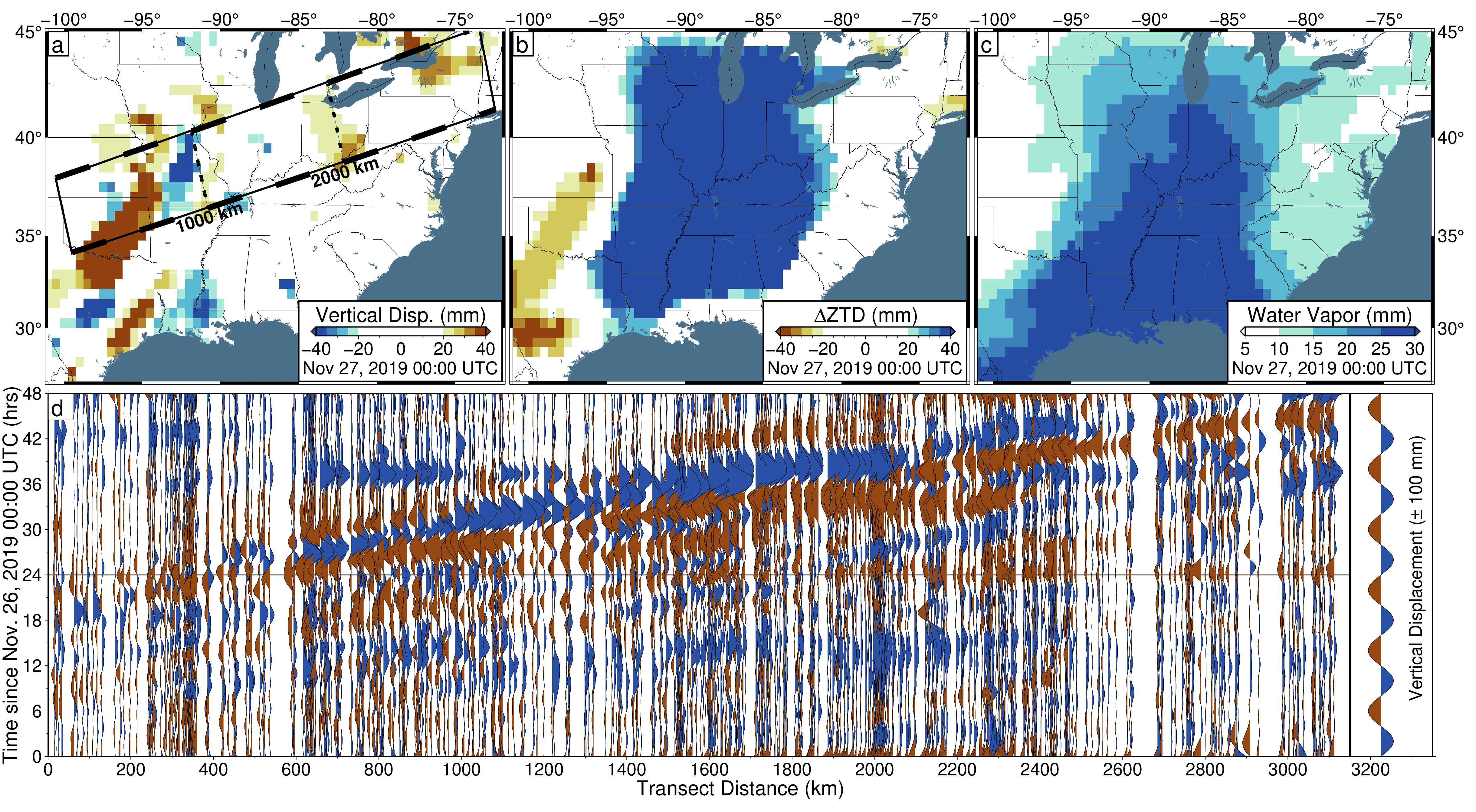


**Video S1:** Observed **(a)** GPS vertical displacement, **(b)** zenith total delay (ZTD) deviation, and **(c)** integrated water vapor, in five minute increments beginning on November 27, 2019 00:00 UTC. Data have been smoothed with Robust Network Imaging and were produced using a random walk of 3 mm/√(hr). **(d)** Wiggle plot of observed vertical displacements for November 26 – 27, 2019. The bounds of the transect are shown by the black rectangle in panel a. Horizontal black line represents the current epoch shown in panels a – c. VLC media player is able to play the videos on most platforms.


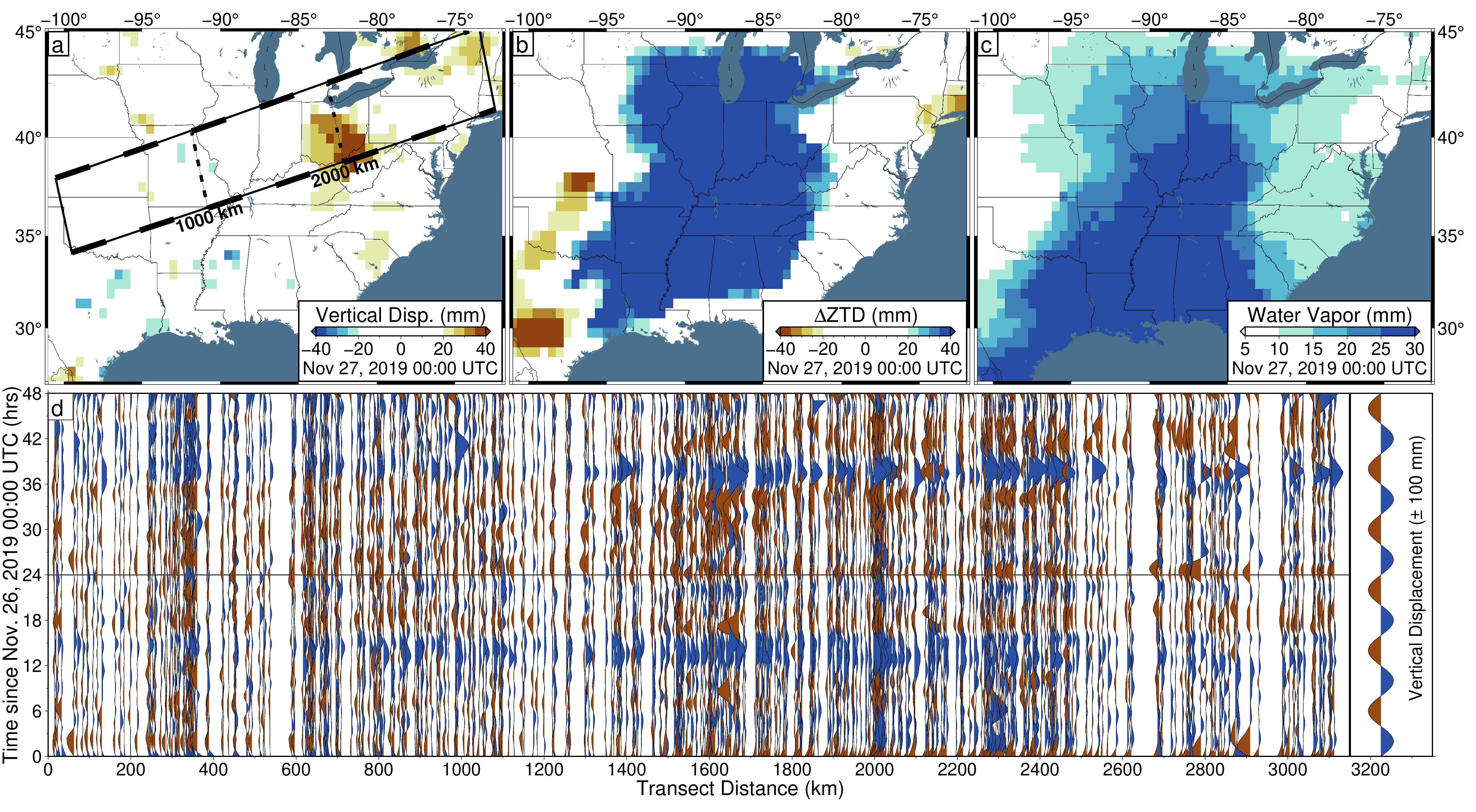


**Video S2:** Same as Video S1, except for the TROPx08 solution.


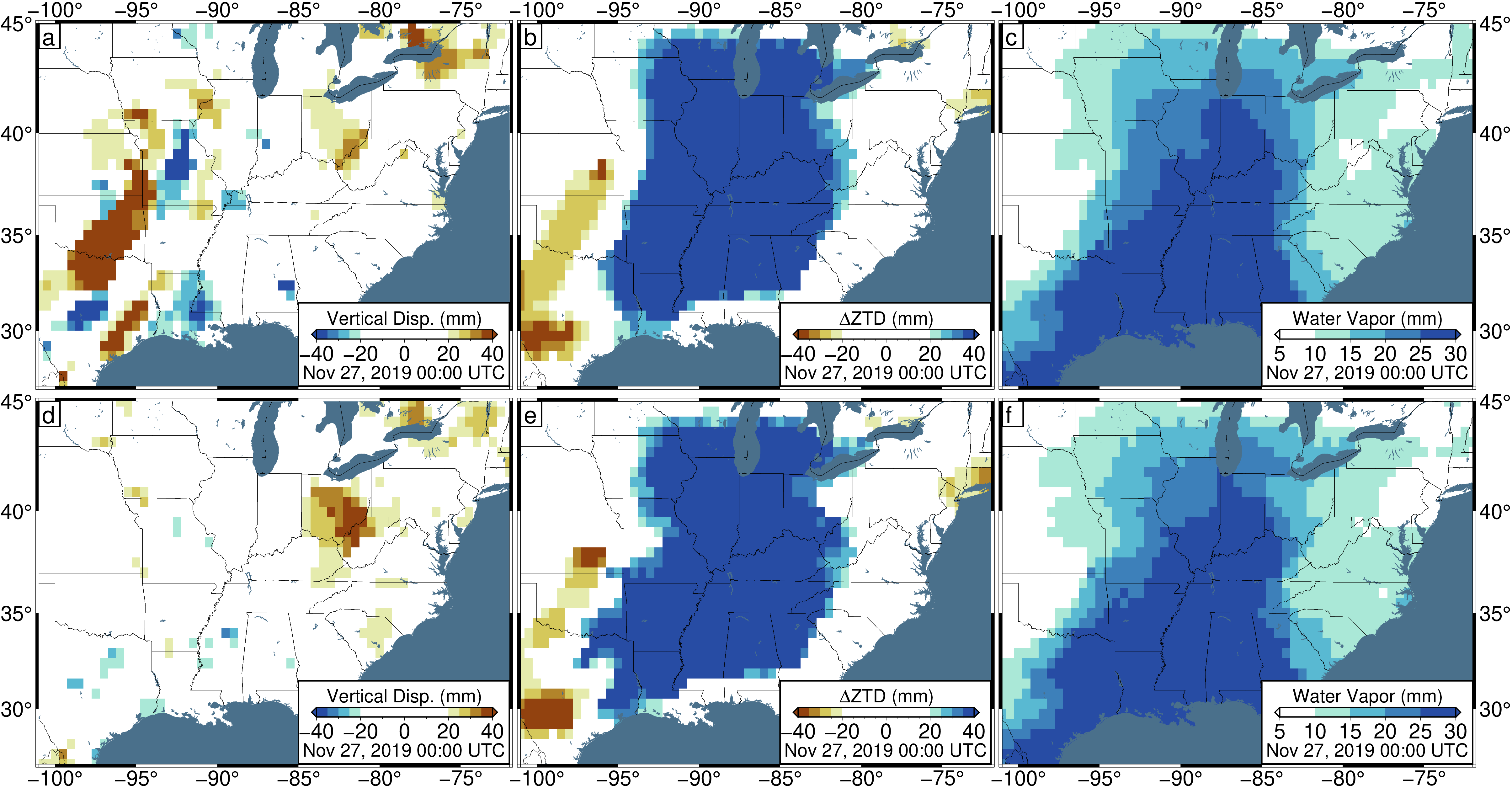


**Video S3:** Direct comparison of the **(a, b, and c)** TROPx01 and **(d, e, and f)** TROPx08 results for **(a and d)** GPS vertical displacement, **(b and e)** zenith total delay deviation, and **(c and f)** integrated water vapor. Key as described in Video 1.
